# Genome sequence of the cauliflower mushroom *Sparassis crispa* (Hanabiratake) and its association with beneficial usage

**DOI:** 10.1038/s41598-018-34415-6

**Published:** 2018-10-30

**Authors:** Ryoiti Kiyama, Yoshiyuki Furutani, Kayoko Kawaguchi, Toshio Nakanishi

**Affiliations:** 1 Department of Life Science, Faculty of Life Science, Kyushu Sangyo Univ., 2-3-1 Matsukadai, Higashi-ku, Fukuoka 813-8503 Japan; 2 Department of Pediatric Cardiology, Tokyo Women’s Medical Univ., 8-1, Kawada-cho, Shinjuku-ku, Tokyo 162-8666 Japan

## Abstract

*Sparassis crispa* (Hanabiratake) is a widely used medicinal mushroom in traditional Chinese medicine because it contains materials with pharmacological activity. Here, we report its 39.0-Mb genome, encoding 13,157 predicted genes, obtained using next-generation sequencing along with RNA-seq mapping data. A phylogenetic analysis by comparison with 25 other fungal genomes revealed that *S*. *crispa* diverged from *Postia placenta*, a brown-rot fungus, 94 million years ago. Several features specific to the genome were found, including the A-mating type locus with the predicted genes for HD1 and HD2 heterodomain transcription factors, the mitochondrial intermediate peptidase (MIP), and the B-mating type locus with seven potential pheromone receptor genes and three potential pheromone precursor genes. To evaluate the benefits of the extract and chemicals from *S*. *crispa*, we adopted two approaches: (1) characterization of carbohydrate-active enzyme (CAZyme) genes and β-glucan synthase genes and the clusters of genes for the synthesis of second metabolites, such as terpenes, indoles and polyketides, and (2) identification of estrogenic activity in its mycelial extract. Two potential β-glucan synthase genes, *ScrFKS1* and *ScrFKS2*, corresponding to types I and II, respectively, characteristic of Agaricomycetes mushrooms, were newly identified by the search for regions homologous to the reported features of β-glucan synthase genes; both contained the characteristic transmembrane regions and the regions homologous to the catalytic domain of the yeast β-glucan synthase gene *FKS1*. Rapid estrogenic cell-signaling and DNA microarray-based transcriptome analyses revealed the presence of a new category of chemicals with estrogenic activity, silent estrogens, in the extract. The elucidation of the *S*. *crispa* genome and its genes will expand the potential of this organism for medicinal and pharmacological purposes.

## Introduction

*Sparassis crispa*, alternatively known as cauliflower mushroom in English or Hanabiratake in Japanese, has been used for food and as a traditional and modern medicine^1^. *S*. *crispa* contains materials with pharmacological activity, such as antitumor, antiangiogenic, antimetastatic, antimicrobial, antiviral, antioxidant, antihypertensive, antidiabetic and antiallergic activities, which are attributable to different effective chemicals with low and high molecular weights such as phenolics and polyphenols, flavonoids and terpenoids^2^. A type of polysaccharide, β-1,3-glucan (β-glucan), which is abundantly obtained from the fruiting body of *S*. *crispa* and has polysaccharide chains linked by β-1,3-glycosidic bonds, has been highlighted because of its potential benefits, such as for prevention of cardiovascular diseases and cancer, due to its immunomodulatory or immunostimulative effects^3^. Immunostimulative activity of β-glucan was commercialized as a drug for the treatment of diseases^4^. However, β-glucan has not been confirmed to be effective for all expected diseases and symptoms, thus chemicals other than β-glucan with respective activities have been considered^2^.

Phytoestrogens, plant-derived chemicals with estrogenic activity, have also been considered as beneficial agents for menopausal syndromes, cardioprotection, neuroprotection and anti-carcinogenesis^5^. Furthermore, chemicals obtained from mushrooms have been used as sources of estrogenic chemicals and have been investigated as alternatives of synthetic estrogens because they may not cause adverse effects or unexpected side effects^6^.

As mushrooms are often misrepresented, it is important to identify medicinal mushrooms at the level of genomic DNA^7^. Here, we report the genomic structure of *S*. *crispa* (strain Scrmy26), and its genes identified by next-generation sequencing and RNA-seq-based transcriptome analysis. We further explored beneficial usages of *S*. *crispa* by two different approaches: finding new β-glucan synthase genes by genome and protein analyses, and identifying new compounds with estrogenic activity by bioassays.

## Results

### Genomic structure and general features

**Figure 1:**
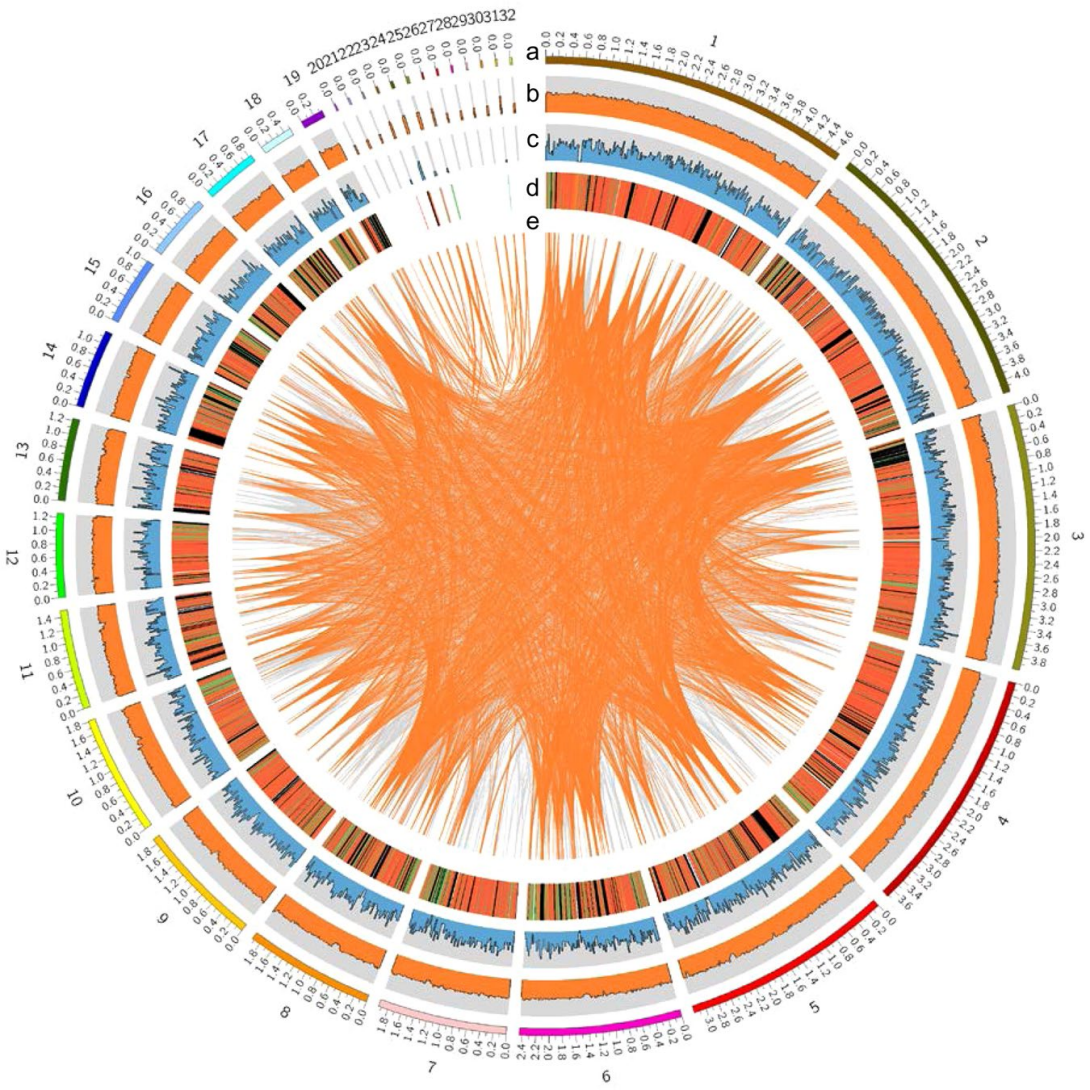
The genomic features of *S*. *crispa*. The ideogram represents (**a**) contigs, (**b**) GC content, (**c**) gene numbers, (**d**) gene expression levels, and (**e**) large segmental duplications. GC content was calculated as the percentage of G + C in 20-kb non-overlapping windows. The abundances of gene transcripts were analyzed as described in Materials and Methods, and expressed in FPKM. The gene number was calculated in 20-kb non-overlapping windows, and the maximum value of the axis was 15. Gene expression levels are shown in red (FPKM > = 100), orange (FPKM > = 10), green (FPKM > 0) and black (FPKM = 0). Large segmental duplications sharing more than 90% sequence similarity are connected by orange (sequence length > = 5 kb) and grey (sequence length > = 2 kb) lines.

The genome of *S*. *crispa* mycelia (strain Scrmy26) was sequenced using a whole genome shotgun sequencing strategy (see Materials and Methods). A 39.0-Mb genome sequence was obtained by assembling approximately 21.3-Gbp reads (>500 × coverage; data not shown) (Table 1). This genome sequence assembly consisted of 32 contigs with an N50 length of 3.18 Mb and L50 of 5 (Fig. 1; Table S1). Based on the number of contigs along with the number of chromosomes expected for mushrooms, we expected the genome size to be close to the obtained size. In total, 13,157 protein-coding genes were predicted, characterized by an average gene length of 1,669.3 bp and average exon number of 5.7 (Table S2). The number of genes in the genome of *S*. *crispa* was comparable with that in genomes of other filamentous fungi^8–14^. The genes predicted formed transcripts with an average length of 1.3 kb and proteins with an average length of 147 amino acids (Table S2). Protein domains are important for the annotation of the genes and proteins identified by the genome analysis^15,16^. We provided here a list of protein domains predicted by the analysis of protein databases (Table S3).

**Table 1:** General features of the *S*. *crispa* genome.

Number of Contigs	32
Length of the genome assembly (Mb)	39.0
GC content (%)	51.4
Number of protein-coding genes	13,157
Average/Median gene length (bp)	1,648.1/1,308
Average/Median protein-coding sequence size (bp)	1,326.1/1,044
Average/Median number of exons per gene	5.7/4
Average/Median exon size (bp)	233.6/137
Average/Median intron size (bp)	73.4/55

Recently, the genome sequence of *S*. *latifolia* was reported^17^, where it showed genome features very close to the data shown here, such as GC contents (51.43% vs 51.42%, our data) and the number of predicted gene models (12,471 vs 13,157), except for a quite difference in the predicted genome sizes (48.13 Mb vs 39.02 Mb). This is probably due to the depth of sequencing, as revealed in the scaffold/contig numbers (472 vs 32) and N50 values (640.83 kb vs 3,179.64 kb). Other features, such as phylogenetic analyses with other fungal genomes and for specific gene functions, did not show much difference, suggesting that reported *S*. *latifolia* and *S*. *crispa* (strain Scrmy26) are very close each other.

### Comparison with other fungal genomes

**Figure 2:**
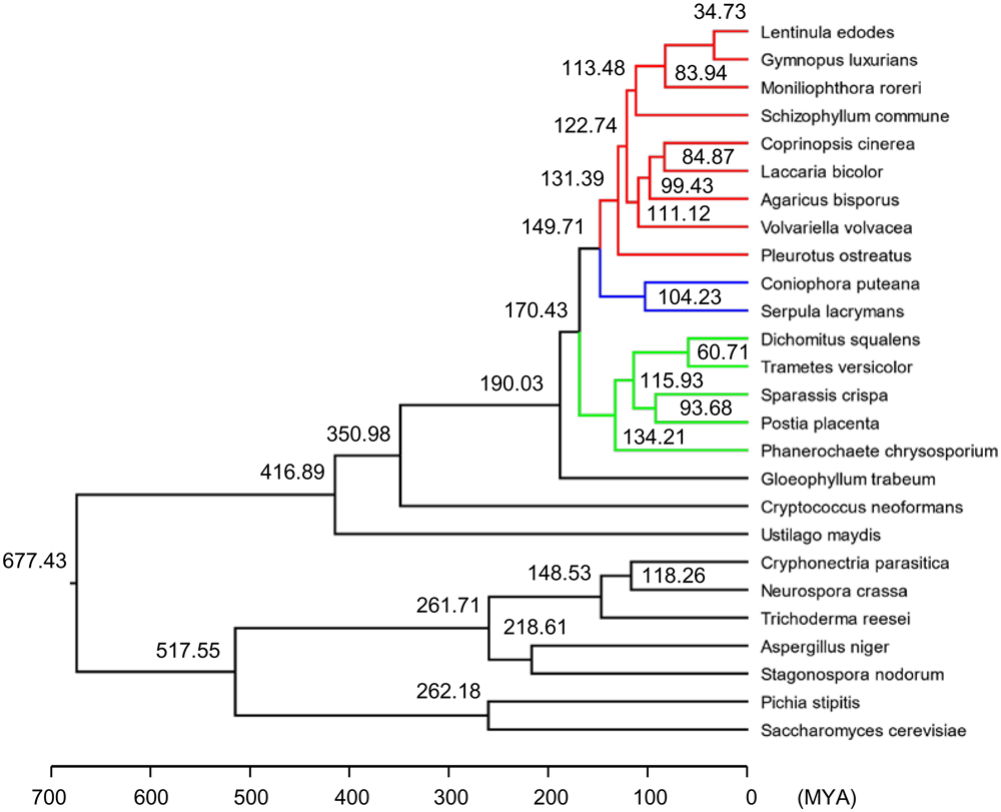
Phylogenetic tree of *S*. *crispa* with 25 other fungal species. The phylogenetic tree was constructed by the maximum likelihood method (see Materials and Methods). MYA: million years ago.

The predicted proteome of *S*. *crispa* was compared with 25 other sequenced fungi (Table S4). The evolutionary history of *S*. *crispa* was examined with a phylogenetic tree (Fig. 2), which was constructed using 895 single-copy orthologous genes conserved in these 26 fungi obtained by OrthoMCL analysis (see Materials and Methods). The molecular clock analysis revealed that *Postia placenta*, a brown-rot fungus^18,19^, was evolutionarily closest fungi to *S*. *crispa*, and their divergence time was estimated to be 94 million years ago (MYA).

### Analysis of mating type loci

**Figure 3:**
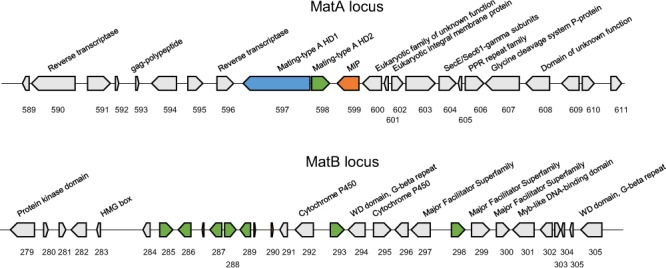
The matA and matB loci of *S*. *crispa*. The genes in the loci are shown. The matA and matB loci are positioned on contigs 1 or 10, respectively.

Two mating type loci, A and B, were identified in the genome sequence of *S*. *crispa* on different contigs (Fig. 3; Table S5). The A-mating-type locus was identified by homology search with the genes for HD1 and HD2 homeodomain transcription factors, and the mitochondrial intermediate peptidase (MIP) of *Coprinopsis cinerea* and *Schizophyllum commune*, where relatively high protein homologies (66~67% identity) were found for MIP, but low homologies (28~34% identity) were found for HD1 and HD2. However, all three genes were found to be located in close proximity on the same contig (contig 1), and there is similarity in the arrangement of HD1 and HD2 genes (located on different strands but with the characteristic outward transcriptional direction) compared with other fungi^20^, suggesting them to be functional. On the other hand, we found a total of seven potential pheromone receptor genes and three potential pheromone precursor genes for the B-mating-type locus, which were mapped on contig 10 (Fig. 3; Table S5).

### CAZymes and glycosyltransferases (GTs)

**Figure 4:**
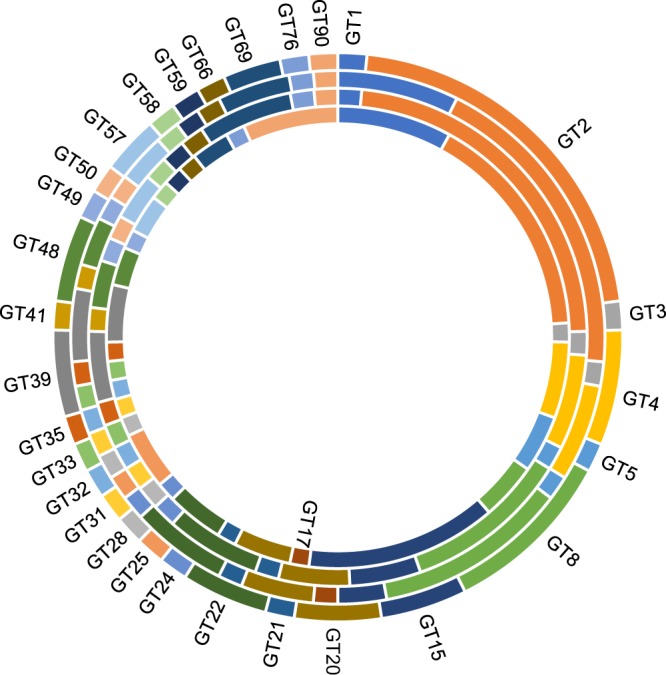
Variation of glycosyltransferases (GTs). *S*. *crispa* (the outer-most ring), *Phanerochaete chrysosporium* (the second ring), *Postia placenta* (the third ring) and *Lentinula edodes* (the inner-most ring).

A total of 246 candidate carbohydrate-active enzyme genes (CAZymes) were identified in the genome of *S*. *crispa*, which included 131 glycoside hydrolases (GHs), 10 carbohydrate esterases (CEs), 61 glycosyl transferases (GTs), 4 polysaccharide lyases (PLs), 19 carbohydrate-binding modules (CBMs) and 31 auxiliary activity enzymes (AAs) (Table S6). The distribution of CAZymes in *S*. *crispa* was compared with 25 other fungi. Compared with the genomes of Agaricales, Polyporales generally had fewer CAZymes, and *S*. *crispa* had the lowest number of GHs among them (Table S6). The variety of GTs (Fig. 4; Table 2) and GHs (Fig. S1; Table 2) of *S*. *crispa* was compared with those of *Phanerochaete chrysosporium*^21^, *Postia placenta*^18^ and *Lentinula edodes*^14^. Compared with other fungi, *S*. *crispa* had the lowest number of genes in each category of CAZymes, and *S*. *crispa*, or the group containing *S*. *crispa*, had the lowest number of GT family genes of GT1, GT5, GT17, GT69 and GT90 (Table S6; Fig. 4). On the other hand, *S*. *crispa* had a low number of GH family genes (about half of that in *Lentinula edodes*), and thus, many GH families had the lowest numbers compared with three other fungi (Table S6; Fig. S1). Thus, *S*. *crispa* may be classified as a fungus with poorly developed carbohydrate utilization ability.

**Table 2:** CAZyme genes in fungi.

CAZyme family	Number of genes			
	*S*. *crispa*	*P*. *chrysosporium*	*P*. *placenta*	*L*. *edodes*
Glycosyl transferase (GT)	61	68	65	75
Glycoside hydrolase (GH)	131	185	144	249
Polysaccharide lyase (PL)	4	6	5	9
Carbohydrate esterase (CE)	10	25	12	30
Carbohydrate-binding module (CBM)	19	61	29	62
Auxiliary activities enzyme (AA)	31	98	49	83
Total	246	403	290	464

### Identification of β-glucan synthases

**Figure 5:**
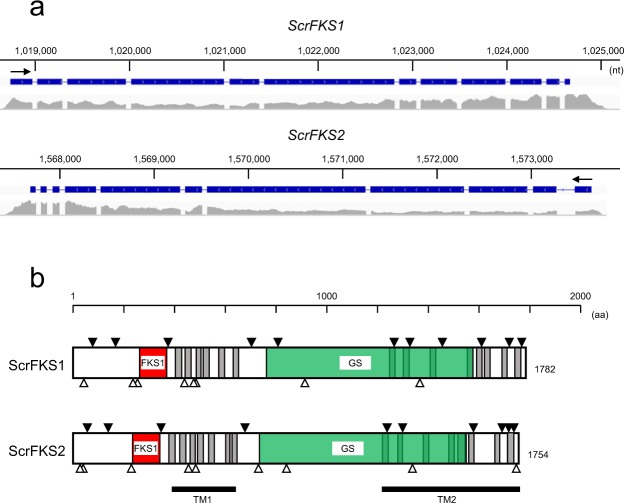
Structure of β-glucan synthases. (**a**) Structure of *ScrFKS1* (on contig 5) and *ScrFKS2* (on contig 4) genes in *S*. *crispa*. The intron-exon structure is shown above, while transcriptome mapping results are shown below. Transcriptional directions are shown by arrows. (**b**) Structure of ScrFKS1 and ScrFKS2 proteins. The transmembrane domains predicted by TMHMM are shadowed, and their clusters are marked with TM1 and TM2. The regions homologous to FKS1 domains (FKS1) and glucan synthases (GS) predicted by Pfam are shown in orange and green, respectively. The potential *N*-glycosylation sites predicted by ScanProsite are shown by open triangles, and the exon-intron junctions are shown by filled triangles.

Among polysaccharides in the fungal cell wall, β-1,3-glucan (β-glucan) is the most prevalent and has been widely used for pharmacological purposes. β-Glucan is synthesized from uridine 5’-diphosphate (UDP)-glucose by β-glucan synthase. β-Glucan synthase is a membrane protein composed of catalytic domains and transmembrane domains. In *Saccharomyces cerevisiae*, there are two independent β-glucan synthase genes (*FKS1* and *FKS2*). Identification of β-glucan synthase genes was performed by BLASTP search with reported genes (see Materials and Methods), where we found two potential β-glucan synthase genes, *ScrFKS1* and *ScrFKS2*, which are homologous to type I and type II genes, respectively (Table S7; Fig. 5). The presence of these two different types is a common characteristic of Agaricomycetes mushrooms^22^. *ScrFKS1* and *ScrFKS2* are homologous to the previously reported type I and type II genes by 77–90% or 77–92%, respectively (Table S7), thereby indicating these to be new genes. Two regions, a region homologous to the catalytic domain of FKS1^23^ and a β-glucan synthase-homologous region, were identified in both genes by Pfam database search, and transmembrane domains by TMHMM database search (Figs 5 and S2). Although two transmembrane domains, TM1 and TM2, are present at identical positions, they differ in that the region between TM1 and TM2 is localized outside the cell in ScrFKS1 but inside in ScrFKS2. In addition, there are several potential *N*-glycosylation sites, some of which are well-conserved between them (Figs 5 and S2); positions 42, 257, 441, 477/478, 912 and 1365 aa (ScrFKS1) and positions 41, 231, 456, 485, 842 and 1338 aa (ScrFKS2), which are conserved between two proteins, are also similar in a sense of the locations relative to the important domains, such as FKS1 and transmembrane domains. Some of them are conserved in another medicinal fungus, *Cordyceps militaris*^24^.

### Biosynthetic gene clusters

Other than highly-polymerized structure of β-glucan, small molecules or secondary metabolites, such as terpenes, indoles and polyketides, have been known to act as bioactive compounds, and the genes responsible for their synthesis are known to form clusters^15,25^. A total of 15 such gene clusters were identified in the *S*. *latifolia* genome, among which the genes encoding terpene synthase for the synthesis of mono-, sesqui-, and di-terpenes, indole prenyltransferase (indole-PTase) or dimethylallyl tryptophan synthase (DMATS) for the synthesis of indole alkaloids, and type I polyketide synthases for the synthesis of aromatic and highly reduced polyketide metabolites, were respectively found^17^. Meanwhile, a total of 30 gene clusters were identified here in the *S*. *crispa* genome (Fig. S3), which include 15, 2, 5 and 8 genes potentially associated with the synthesis/metabolism of terpenes, indoles, polyketides and others, respectively (summarized in Table S8). Note that all these chemical categories have been known to include chemicals with estrogenic activity and that some are derived from fungi, such as zearalenone and brefeldin A^26^. Thus, we next tried to find chemicals with estrogenic activity in the extract of *S*. *crispa*.

### Estrogenic activity associated with the mycelial extract

**Figure 6:**
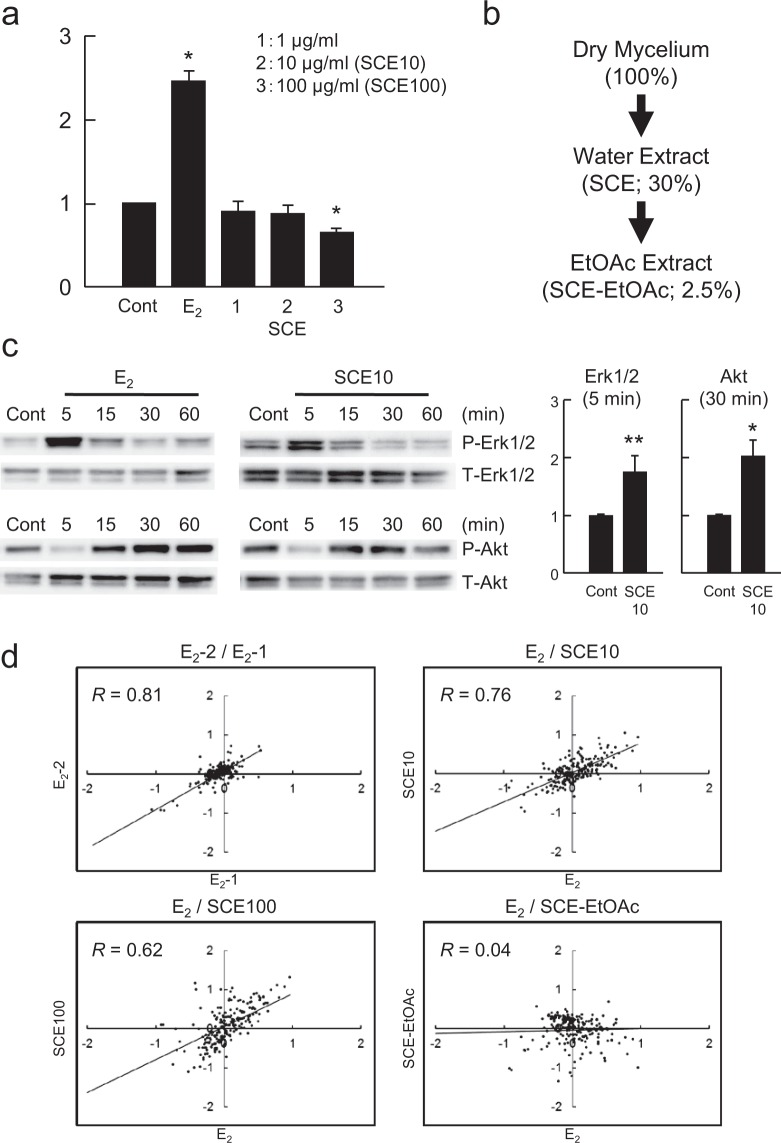
Estrogenic activity of *S*. *crispa* mycelial extracts. (**a**) Cell proliferation assay. MCF-7 cells were treated with vehicle (DMSO), E_2_ (10 nM) or the extracts as indicated. Cell proliferation was examined by sulforhodamine B (SRB) assay. The ratio of cell proliferation rate in response to E_2_ or the extracts to that to the control (DMSO) is shown in the graphs. *p < 0.01 vs. control (Cont). (**b**) The extraction strategy and the yields (%) of materials (dry weight). (**c**) Western blot analysis of the activation of Erk1/2 and Akt. MCF-7 cells were treated with 10 nM E_2_ or SCE10 for the indicated times (min), and cell extracts were subjected to Western blot analysis for phosphorylated (P-) or total (T-) proteins as indicated (*left*). The results of three independent experiments are summarized along with statistical evaluation in the graphs (*right*). Significance of data compared with the controls was evaluated using *p*-values of *p < 0.01 or **p < 0.05. (**d**) The gene expression profiles for these chemicals were compared using a set of 150 estrogen-responsive genes in scatter-plot graphs. The vertical and horizontal axes indicate log_2_ values of the signal intensities. *R*-values were calculated for each graph on the basis of linear regression between two profiles.

We then examined whether the water extract of *S*. *crispa* (SCE) contains beneficial components by screening for silent estrogens, which has been reported for an edible mushroom *Agaricus blazei* (*A*. *blazei*)^27^. A silent estrogen is a category of chemicals that exhibit estrogenic gene expression profiles but do not have any growth-stimulating activity in estrogen-responsive breast cancer MCF-7 cells, and thus may be beneficial for preventing cancer progression and as alternatives for estrogen antagonists^28^. SCE did not exhibit any growth-stimulating activity in MCF-7 cells (Fig. 6a), but it rapidly activated both Erk1/2 and Akt (Fig. 6c), which are markers of the non-genomic pathway of estrogen signaling^29^. We also examined whether the activity was retained after extraction of SCE with ethylacetate (Fig. 6b), which was the case for *A*. *blazei*^30^. No estrogenic activity was detected by DNA microarray-based gene expression profiling for the ethylacetate extract (*R* = 0.04, Fig. 6d), but SCE had profiles (*R* = 0.76 for SCE10 or 0.62 for SCE100) comparable with that for 17β-estradiol (E_2_) (*R* = 0.81). As SCE contained very small amounts of β-glucans (0.95% of the amount in the dry mycelium, data not shown), the chemical(s) responsible for estrogenic activity may be a water soluble chemical with a low molecular weight.

## Discussion

*S*. *crispa* is a brown-rot fungus that grows primarily on the stumps of coniferous trees, and is widely distributed throughout the North Temperate Zone^2^. *S*. *crispa* has been known as a source of natural products with bioactive properties, such as for nutraceutical^31^, medicinal^32,33^ and cosmetic^34^ applications. Among compounds found in *S*. *crispa*, β-glucan has been extensively studied as chemicals effective for neuroprotection^35^, cardioprotection^36^ and anti-inflammation^37^. In contrast, recent studies showed that the aromatic compounds isolated from *S*. *crispa* are effective for the treatment of cancer^38^ or hyperlipidemia^39^. Here, we used the mycelium of *S*. *crispa* (strain Scrmy26) as a source both for the genome/gene analysis and the analysis of bioactive materials to employ a consistent system for the analyses. We focused on the mycelium here because controlling the culture conditions would be easier and thus mass production and quality control could be standardized. Furthermore, *S*. *crispa* is one of the mushrooms that contain the highest phenolic contents and the highest antioxidant capacity in mycelia^40^.

The analysis of the *S*. *crispa* genome revealed its relatively high GC% (51.4%; Table 1) among the 26 fungal genomes compared here, while the sizes of the genome and the number of protein-coding genes are well within their variations. From the phylogenetic analysis, *S*. *crispa* was likely diverged from *Postia placenta* 94 MYA. Both *S*. *crispa* and *P*. *placenta* belong to brown-rot fungi, which degrade plant cell walls by ligninolysis with distinct differences from while-rot fungi including the mechanism of lignocellulose conversion^18^. The numbers of categories of CAZyme genes, such as GHs and GTs, are comparable between them (131 GHs for *S*. *crispa* and 144 GHs for *P*. *placenta*, or 61 GTs for *S*. *crispa* and 65 GTs for *P*. *placenta*; Table S6), suggesting similarity in their lifestyle evolutions^41^. As the classification of fungi has often been controversial and, several revisions have resulted in several taxonomic synonyms for *P*. *placenta*^42^, it will be quite informative to find the similarity as well as the differences in genome sequences between these two fungi to understand their lifestyles.

A total of 5,601 genes were annotated by Gene Ontology database, which include 1,562, 4,931 or 3,437 genes related to cellular components, molecular functions or biological processes, respectively (Fig. S4). Significant numbers of genes were found to be associated with cell/cell part/organelle/membrane (cellular component), binding/catalytic activity (molecular function), and metabolic process/cellular process (biological process). Meanwhile, a total of 6,460 genes were classified into the pathways by the Kyoto Encyclopedia of Genes and Genomes (KEGG) database, which were further classified into the metabolic pathways (1,753 genes), and the pathways related to genetic information processing (951), environmental information processing (576), cellular processes (852), organismal systems (1,011) and diseases (1,317) (Fig. S5). A significant number of genes (465) were related to carbohydrate and glycan metabolisms, including 37 genes for glycolysis/gluconeogenesis and 48 genes for *N*-glycan biosynthesis, suggesting these genes to be heavily utilized for the survival of the fungus.

Polysaccharides, which account for over 90% of fungal cell walls, are predominantly β-glucans, with the interchain composed of mainly β-1,3- and 3-4% β-1,6-glucosidic linkages^43^. There are two identifiers for the search of β-glucan synthase (EC 2.4.1.34), Glucan_synthase (PF02364) and FKS1_dom1 (PF14288), and two GT families including β-glucan synthase, GT2 and GT48, in the CAZy database. The database search resulted in the identification of two potential genes for β-glucan synthase, *ScrFKS1* and *ScrFKS2* (Figs 5 and S2), which are likely the genes for type I and type II genes characteristic to Agaricomycetes mushrooms^22^, respectively. Although the details are not yet known, there two types may have differences in catalytic activity and function in different developmental stages^22^. There are significant similarities in domains and motifs between ScrFKS1 and ScrFKS2 (see Fig. 5b), where we observed conservation of transmembrane domains, the catalytic FKS1 domain and *N*-glycosylation sites.

Hot water extraction of *S*. *crispa* powder resulted in the yield of β-glucan to be 50.6%^32^, although the activity of rapid estrogenic signaling through Erk1/2 and Akt was detected in the fraction of low β-glucan contents (Fig. 6c). The same extract revealed expression profiles similar to those after the treatment with E_2_ (Fig. 6d), suggesting the activity to be estrogenic. However, the extract failed to promote cell proliferation (Fig. 6a), thereby categorizing the activity to be that by silent estrogens. Silent estrogens are a group of chemicals with estrogenic gene-expression profiles but lacking estrogen-induced cell proliferation^28^, and include chemicals such as brefeldin A^30^ and capsaicin^44^, and the mixtures of chemicals, such as the degradation product of crude oil^45^. While phenolics are supposed to have a main structural characteristic for estrogenic chemicals, there are a number of exceptions in the structure responsible for the activity^26^, enlarging the potential of activities other than the promotion of cell proliferation, and the chemicals with such a characteristic would form a quite important category of bioactive materials.

## Materials and Methods

### Materials

*S*. *crispa* powder was prepared from mycelia of *S*. *crispa*, strain Scrmy26, which was supplied by Intertrade Co., LTD. The genomic DNA of *S*. *crispa* was prepared with a NucleoBond buffer Set III kit (Macherey-Nagel, Düren, Germany). The DNA library for genome sequencing was constructed with 28 μg of purified DNA with the SMRTbell Template Preparation Kit 1.0 and DNA/Polymerase Binding Kit P6 (Pacific Biosciences; Menlo Park, CA).

### DNA sequencing and genome assembly

The *S*. *crispa* genome was sequenced by Hokkaido System Science Co., Ltd. (Sapporo, Japan), using 16 SMRT cells on the PacBio RSII platform (Pacific Biosciences) with P6-C4 chemistry, generating 21.3 Gbp (>500 × coverage). The mean, N50, and the longest insert read length of the generated data were 9,616 bp, 13,871 bp and 52,687 bp, respectively. The obtained reads were assembled using Falcon pipeline with slight modifications to the predefined parameter set distributed by the developers^46^. The assembled contigs were polished using the Arrow algorithm in the GenomicConsensus software package (https://github.com/PacificBiosciences/GenomicConsensus). The genome sequence data are available in the DDBJ BioProject Database of the DNA Database of Japan (DDBJ: http://www.ddbj.nig.ac.jp/) under Accession No. BFAD01000001-BFAD01000032 (PRJDB5582).

### rRNA and tRNA identification

rRNAs were identified by Barrnap (https://github.com/tseemann/barrnap), a ribosomal RNA predictor. tRNAscan-SE^47^ was used to detect tRNA regions and secondary structures.

### Gene expression analysis

A cDNA library was prepared from the total RNA using the TruSeq Stranded mRNA Sample Prep Kit (Illumina, San Diego, CA), and subjected to sequencing on the Illumina HiSeq 2500 platform. Approximately 80 million reads were obtained and used for genome annotation and gene expression analysis. The expression level of each gene was quantified by FPKM (expected fragments per kilobase of transcript per million fragments sequenced) values using Cufflinks^48^ software as follows. After adapter sequences were removed from RNA-seq reads using CutAdapt^49^, the primary contigs of the assembly were mapped using TopHat2^50^. Finally, the mapped reads were counted for each gene model and reported in FPKM using Cufflinks.

### Protein-coding gene prediction and functional annotation

The prediction of protein-coding genes along with their functional annotation was assisted by GeneBay Inc. (Yokohama, Japan). The RNA-seq reads were first aligned to the reference genome with HISAT^51^, then assembled by StringTie^52^ to construct transcript sequences. The obtained 15,184 transcripts were used to train AUGUSTUS^53^ for parameters of gene models in *S*. *crispa*. AUGUSTUS was run with exon and intron hints, derived from RNA-seq mapping data, as input data. On the other hand, CodingQuarry^54^, which was developed to accurately predict genes in fungal genomes, was used to predict gene models using the above StringTie-derived transcript sequences. The gene models predicted by AUGUSTUS, CodingQuarry and StringTie were combined to construct 13,156 consensus gene models using EvidenceModeler^55^. All of the predicted gene models were functionally annotated based on the similarity to annotated genes. BLASTP^56^ was used to align the protein sequences to Nr and fungal taxonomic divisions of Swiss-Prot^57^ protein databases with an e-value < 1e-5.

### Phylogenetic analysis

A total of 26 fungal species assigned to *Basidiomycota* or *Ascomycota*, including *S*. *crispa*, were used in the phylogenetic analysis. Their protein sequences were compared by BLASTP (e-value < 1e-5 and hit number < 500). The BLASTP result was then analyzed by OrthoMCL^58^ with default parameters to find orthologous genes, where a total of 895 single-copy orthologous genes were determined. The sequence alignments of these 895 genes were calculated by MAFFT v7.309^59^, and then combined into a long sequence for each species. The conserved blocks of the alignment were selected by Gblocks 0.91b^60^ with the default parameters. Finally, the phylogenetic tree was constructed by RAxML-8.2.9^61^ with bootstrap 1000. The fossil calibration was done as described^62^ by fixing the following three points in the molecule clock analysis: the most recent common ancestor (MRCA) of *Coprinopsis cinerea*, *Laccaria bicolor* and *Schizophyllum commune* diverged at 122.74 MYA; the MRCA of *Serpula lacrymans* and *Coniophora puteana* diverged at 104.23 MYA; and the MRCA of *Pichia stipitis*, *Aspergillus niger*, *Cryphonectria parasitica*, *Stagonospora nodorum* and *Trichoderma reesei* diverged at 517.55 MYA. The divergence time of other nodes was then calculated by r8s v1.81^63^ with the TN algorithm, PL method and the smoothing parameter value set to 1.8.

### Identification of matA and matB genes

The matA genes were identified by the similarity with the matA and MIP genes of *Coprinopsis cinerea* and *Schizophyllum commune*. The pheromone receptor genes were identified by the search with key word “pheromone receptor” in Swiss-Prot. The sequences of pheromone precursors, usually 50~60 aa in length, were searched in the ~20 kb flanking region of the pheromone receptor genes by Transdecoder (https://transdecoder.github.io/) with Pfam search (https://pfam.xfam.org/).

### Protein domain analysis and gene function classification

The amino acids sequences translated from the predicted gene models were analyzed using InterProScan (5.26–65.0) to annotate functional domains by the Pfam database (https://pfam.xfam.org/)^64^ as well as to assign Gene Ontology (GO) categories (http://www.geneontology.org/)^65^. The assigned GO terms are then mapped to the plant subset of GO categories (GO Slims) using Map2Slim script in the OWLTools (https://github.com/owlcollab/owltools). Protein domains are provided in the *S*. *crispa* genome sequence database (see above). Gene function pathways were analyzed by submitting BLAST data to KEGG (http://www.genome.jp/kegg/)^66^ database. The Gene Ontology data were visualized by WEGO (http://wego.genomics.org.cn/)^67^. Biosynthetic gene clusters in the *S*. *crispa* genome were identified by antiSMASH (http://antismash.secondarymetabolites.org/)^25^.

### Identification of CAZymes and β-glucan genes

Carbohydrate-active enzymes (CAZymes) were classified by HMM search with dbCAN HMMs 5.0^68^ (under the default cutoff threshold) and BLASTP search of CAZy database^69^ (e-value < = 1e-6; the covered fraction ratio > = 0.2; the maximum hit number = 500). The BLASTP results screened with the threshold used in the study of *L*. *edodes*^14^ were then added to the common results to obtain the final CAZyme annotation. Meanwhile, β-glucan genes were identified by BLAST search with reported genes for type I and II genes^22^, and the obtained sequences were analyzed for catalytic domains and transmembrane regions by searching Pfam or TMHMM (http://www.cbs.dtu.dk/services/TMHMM/) databases.

### Preparation of *S*. *crispa* mycelial extracts

The aqueous extract of *S*. *crispa* mycelia (SCE) was prepared by boiling 50 g of the *S*. *crispa* powder in 1,000 ml of Milli-Q water for 10 min. The aqueous fraction was recovered by centrifugation at 3,300 × g for 25 min and sterilized by filtration through 0.22-μm filters (Millipore; Billerica, MA). To prepare the ethylacetate fraction (SCE-EtOAc), 100 ml of 10 mg/ml SCE was mixed with 100 ml of ethylacetate, and SCE-EtOAc was recovered as the upper fraction after centrifugation at 7,000 × g for 10 min. SCE-EtOAc was then dried by evaporation. The dried material dissolved in methanol was centrifuged at 3,300 × g for 10 min at 25 °C to remove debris, and the final concentration was adjusted to 10 mg/ml.

### Western blotting

MCF-7 cells were obtained from the JCRB Cell Bank (National Institute of Health Sciences, Tokyo, Japan), and cultured in RPMI 1640 (Invitrogen, Carlsbad, CA) medium supplemented with 10% fetal bovine serum (FBS) at 37 °C in 5% CO_2_. Western blotting was performed according to the protocol described in Dong *et al*.^30^. Phospho-Erk1/2 (P-Erk1/2) and phospho-Akt (P-Akt) were used as the primary antibodies, and rabbit antibodies against total Erk1/2 (T-Erk1/2), P-Erk1/2, total Akt (T-Akt) or P-Akt (Cell Signaling Technologies, Ipswich, MA) were used as the secondary antibodies after appropriate dilutions (1:200 to 1:1000). The antibody-antigen complexes were detected with horseradish peroxidase-coupled goat antibody against rabbit IgG (Cell Signaling Technologies) after dilution (1:3,000), and then visualized using the ECL-plus Western Blotting Detection System (Amersham Pharmacia Biotech, Arlington Heights, IL).

### Cell proliferation assay

The cell proliferation assay was performed as described in Dong *et al*.^30^. MCF-7 cells were cultured in phenol red-free RPMI 1640 medium supplemented with 10% dextran-coated charcoal-treated fetal bovine serum (DCC-FBS, Invitrogen) in 24-well plates. The cells were treated with 10 nM E_2_ (17 β-estradiol) or the extracts under the indicated concentrations for 3 days. The cells were then fixed with 10% cold trichloroacetic acid (TCA) at 4 °C for 30 min and stained with 0.4% sulforhodamine B (SRB) dissolved in 1% acetic acid for 20 min. The bound protein was solubilized with 10 nM unbuffered Tris-base and subjected to measurement of OD_490_. Six independent assays were performed for each treatment and the data were analyzed by the *t*-test.

### DNA microarray assay

MCF-7 cells were maintained in phenol red-free RPMI 1640 medium containing 10% DCC-FBS, and incubated for 3 days at 37 °C in 5% CO_2_. Aliquots of E_2_ (10 nM), SCE (10 or 100 μg/ml) or SCE-EtOAc (10 μg/ml) in 0.1% DMSO were added to the medium, and the cells were cultured for 3 days. Cells treated with 0.1% DMSO (vehicle) were used as a control. RNA preparation and cDNA labeling followed by focused oligonucleotide-DNA microarray assays were performed as described previously^27,70^. The values obtained were normalized and log_2_-transformed, and then used for correlation analysis. A coefficient of correlation between gene expression profiles was calculated based on linear regression using SPSS 12.0J (SPSS Japan; Tokyo, Japan). We here used a total of 150 genes selected as highly reproducible estrogen-responsive genes as described before^27^. The microarray data are available in the Gene Expression Omnibus database of the National Center for Biotechnology Information (http://www.ncbi.nlm.nih.gov/geo/) under Accession No. GSE108549.

## Electronic supplementary material

Supplementary Information

Supplementary Table S3

Supplementary Table S6
