# Supplementary material for: Genome sequence of the cauliflower mushroom Sparassis crispa (Hanabiratake) and its association with beneficial usage
Source: Sci Rep. 2018 Oct 30;8:16053. doi: 10.1038/s41598-018-34415-6 (PMC6207663; doi:10.1038/s41598-018-34415-6)
Supplement: Supplementary file 1 — Supplementary Information [file 41598_2018_34415_MOESM1_ESM.pdf]

Supplementary Information

Genome sequence of the cauliflower mushroom *Sparassis crispa* (Hanabiratake) and its association with beneficial usage

Ryoiti Kiyama [1*], Yoshiyuki Furutani [2], Kayoko Kawaguchi [2], Toshio Nakanishi [2]
[1] Dept. of Life Science, Faculty of Life Science, Kyushu Sangyo Univ., 2-3-1 Matsukadai, Higashi-ku, Fukuoka 813-8503, Japan.
[2] Dept. of Pediatric Cardiology, Tokyo Women's Medical Univ., 8-1, Kawada-cho, Shinjuku-ku, Tokyo 162-8666, Japan.
Correspondence and requests for materials should be addressed to R. K. (email: kiyama.r@ip.kyusan-u.ac.jp)

Supplementary Information

Figure S1. Variation of glycoside hydrolases (GHs). See Fig. 4 for details.

Figure S2. Nucleotide sequences and deduced amino acid sequences of *S. crispa* β-glucan synthases.
Nucleotide sequences of *S. crispa* β-glucan synthase genes, *ScrFKS1* (a) and *ScrFKS2* (b), and the deduced amino acid sequences are shown. The transmembrane domains predicted by TMHMM are shadowed, the regions homologous to FKS1 domains and glucan synthases predicted by Pfam are indicated by solid or dashed lines, respectively (a and b), and the potential *N*-glycosylation sites predicted by ScanProsite are boxed (a and b). The exon-intron junctions are shown by triangles (a and b).

Figure S3. Biosynthetic gene clusters in the *S. crispa* genome.
The genes with predicted functions are: core biosynthetic genes (■), additional biosynthetic genes (■), transport-related genes (■), regulatory genes (■) and other genes (■).

Figure S4. Results of Gene Ontology classification.

Figure S5. Results of KEGG pathway classification.

Table S1. Assembly statistics.

Table S2. Gene model statistics.

Table S3. List of protein domains found in the genes of *S. crispa*.

Table S4. Resources of the other 25 fungi for OrthoMCL analysis.

Table S5. **The loci of matA and matB genes.**

Table S6. **Distribution of CAZyme genes in *S. crispa* and the other 25 fungi.**

Table S7. **Identification of type I and type II β-glucan synthase genes in *S. crispa*.**

Table S8. **List of biosynthetic gene clusters in the *S. crispa* genome.**

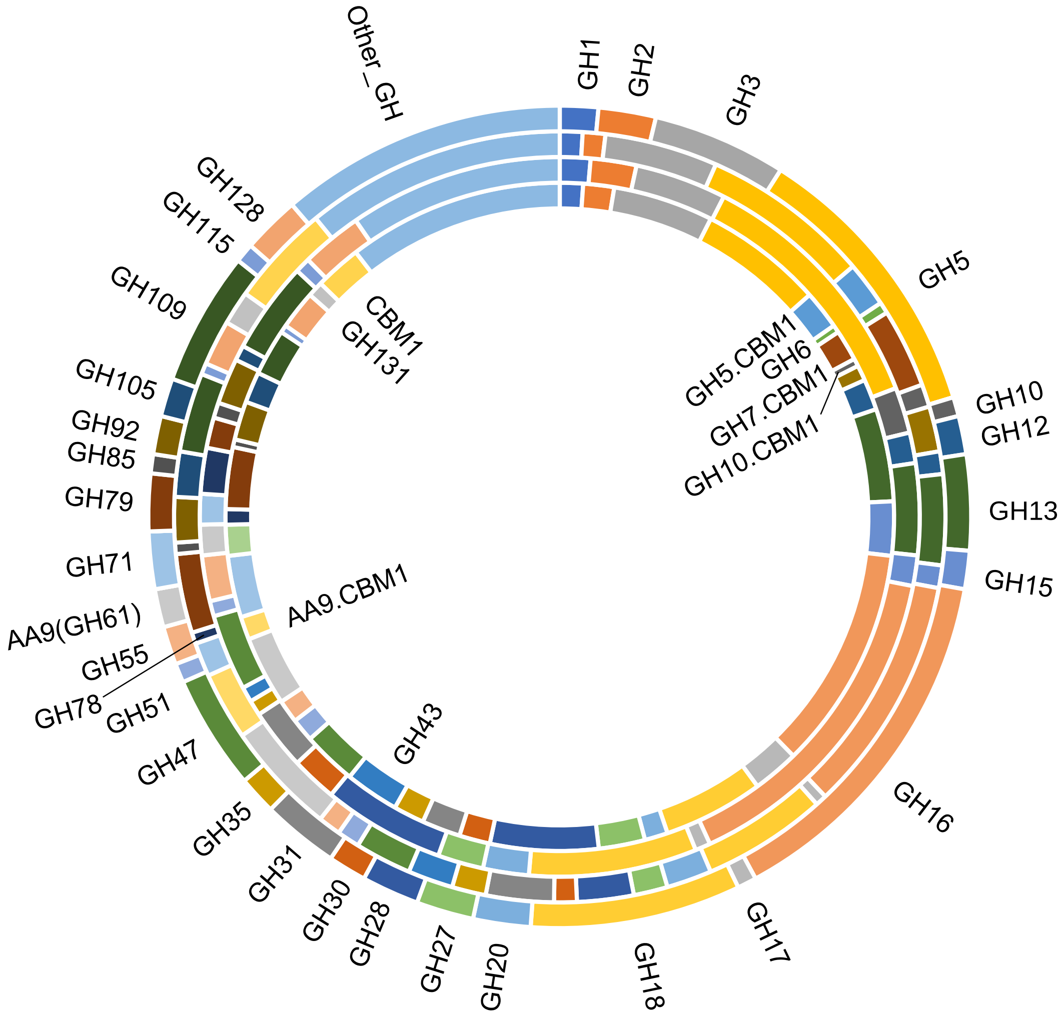

Other_GH
GH1
GH2
GH3
GH5
GH10
GH12
GH13
GH15
GH16
GH17
GH18
GH20
GH27
GH28
GH30
GH31
GH35
GH47
GH51
GH78
GH55
AA9(GH61)
GH71
GH79
GH85
GH92
GH105
GH109
GH115
GH128
CBM1
GH131
GH5.CBM1
GH6
GH7.CBM1
GH10.CBM1
AA9.CBM1
GH43

Fig. S1.

Fig. S2. Nucleotide sequences and deduced amino acid sequences of *S. crispa* β-glucan synthases. Nucleotide sequences of *S. crispa* β-glucan synthase genes, *ScrFKS1* (a) and *ScrFKS2* (b), and the deduced amino acid sequences are shown. The transmembrane domains predicted by TMHMM are shadowed; the regions homologous to FKS1 domains and glucan synthases predicted by Pfam are indicated by solid or hatched lines, respectively (a and b); and the potential *N*-glycosylation sites predicted by ScanProsite, are boxed (a and b). The exon-intron junctions are shown by triangles (a and b).

(a)

```
  1   ATGGCTGGTCGCCCCGGTCCACCTCGCAGCCATGAGGGCATCTACGTTTCCCGCTCGCCT
  1   M  A  G  R  P  G  P  P  R  S  H  E  G  I  Y  V  S  R  S  P
 61   TCTTCTGATCCCCACGATCCCTTCAGCAACGGGCAAGCGGCGGACGCACAGAGGTACTAC
 21   S  S  D  P  H  D  P  F  S  N  G  Q  A  A  D  A  Q  R  Y  Y
121   GATAACGACTCTGACAACTATGGCCGTCGCGACACCTACATGTCAGACGGCAGCAATACT
 41   D  N  D  S  D  N  Y  G  R  R  D  T  Y  M  S  D  G  S  N  T
181   GGTCTGAATGAGGAGGATCGCTATTACGACAATAATGGTTACGATGCGTACACGCAACCC
 61   G  L  N  E  E  D  R  Y  Y  D  N  N  G  Y  D  A  Y  T  Q  P
241   GATACCGATTCGGAGGTTGATGTGTACGGCCAGAAATATGCACCCTCTGCCGAATCGCTG
 81   D  T  D  S  E  V  D  V  Y  G  Q  K  Y  A  P  S  A  E  S  L
301   GGCCCCCCTCGCATGGGTCTTTCAGAAGCATCTACTCCCACTTTTGTCGACCAGAATGGC
101   G  P  P  R  M  G  L  S  E  A  S  T  P  T  F  V  D  Q  N  G
361   GGTCCTCCAGGCTCACGCGAGCCGTACCCTGCGTGGACATCGGAAAGACAAATTCCCTTG
121   G  P  P  G  S  R  E  P  Y  P  A  W  T  S  E  R  Q  I  P  L
421   TCGAAGGAGGAGATTGAGGACATTTTTCTCGACCTGACCCAGAAGTTCGGCTTCCAACGA
141   S  K  E  E  I  E  D  I  F  L  D  L  T  Q  K  F  G  F  Q  R
481   GACTCCATGCGCAACATGTTCGACTTCACCATGCAGCTGCTGGACAGTCGTGCCTCCCGC
161   D  S  M  R  N  M  F  D  F  T  M  Q  L  L  D  S  R  A  S  R
541   ATGTCGCCAAATCAAGCCCTTCTCACGCTGCACGCAGACTACATCGGTGGTCAGCATGCC
181   M  S  P  N  Q  A  L  L  T  L  H  A  D  Y  I  G  G  Q  H  A
601   AACTACCGCAAGTGGTACTTCGCTGCGCAGCTTGACCTCGACGATGCAGTCGGGCACACA
201   N  Y  R  K  W  Y  F  A  A  Q  L  D  L  D  D  A  V  G  H  T
661   CAAAATCCTGGTCTGCAACGCCTGAAGTCGACGAAACGTGGTGGCCATAGGAACAATTCA
221   Q  N  P  G  L  Q  R  L  K  S  T  K  R  G  G  H  R  N  N  S
721   GAGAAGTCGCTTAATAGTGCGATGGACCGATGGCGGCAGGCGATGAACAACATGAGCCAG
241   E  K  S  L  N  S  A  M  D  R  W  R  Q  A  M  N  N  M  S  Q
781   TATGATCGCATGCGCCAGATCGCGCTCTTTCTGCTGTGCTGGGGAGAAGCCGCACAGGTG
261   Y  D  R  M  R  Q  I  A  L  F  L  L  C  W  G  E  A  A  Q  V
841   CGGTTCGTGCCCGAGTGTCTCTGCTTCATCTTCAAGTGTGCGGATGATTATTATCGGTCG
281   R  F  V  P  E  C  L  C  F  I  F  K  C  A  D  D  Y  Y  R  S
```

901 CCGGAGTGTCAGAACAAGATCGATCCGGTGCCTGAGAGCTTGTACCTTCGTACCATCGTC

301 P E C Q N K I D P V P E S L Y L R T I V

961 AAGCCTTTGTATACGTTCATTCGGGACCAGGGATATGAGGTAGTCGATGGGAAGTTCGTC

321 K P L Y T F I R D Q G Y E V V D G K F V

1021 CGCCGGGAGAAGGATCACGAGAGCATCATCGGTTATGACGACGTCAATCAGCTCTTCTGG

341 R R E K D H E S I I G Y D D V N Q L F W

1081 TACCCGGAGGGCATTGCTCGTATTGTACTAACCGACAAGACTCGGTTGGTTGATCTGCCG

361 Y P E G I A R I V L T D K T R L V D L P

1141 CCCGCGCAACGATTCATGAAGTTTGAGCGAATCGACTGGAACCGTGTGTTCTTCAAGACG

381 P A Q R F M K F E R I D W N R V F F K T

1201 TACTACGAGAAGCGATCGTTTGGACACCTGTTGGTCAACTTCAATCGGATCTGGGTCATT

401 Y Y E K R S F G H L L V N F N R I W V I

1261 CACATCGCCATGTACTGGTTCTATACGGCGTACAACTCGCCGACCATCTATAATGGGCAG

421 H I A M Y W F Y T A Y N S P T I Y N G Q

1321 AACTCTTCAGCGATGAGATGGAGTGCGACAGCTCTCGGTGGTGCGGTTGCGACAATAATT

441 N S S A M R W S A T A L G G A V A T I I

1381 ATGATTGCCGCTACACTGGCCGAATTCTCGTACATCCCCACCACCTGGAACAACACTTCT

461 M I A A T L A E F S Y I P T T W N N T S

1441 CACCTTACCCGGCGTTTACTTTTCCTCATCGTCACGTTGGCTCTCACGGGTGGGCCGACA

481 H L T R R L L F L I V T L A L T G G P T

1501 ATCTATGTCGCGATCGCAGAGAACTCGTCACCTGGCGGGTCGTTGGCGCTCATCCTCGGT

501 I Y V A I A E N S S P G G S L A L I L G

1561 ATTGTCCAGTTCTTCATCTCTGTCGTCGCCACTCTCCTCTTCGCCATTGTCCCATCAGGT

521 I V Q F F I S V V A T L L F A I V P S G

1621 CGTATGTTCGGTGATAGAGTTGCCGGCAAGTCGCGGAAGTACCTCGCCAGCCAGACCTTC

541 R M F G D R V A G K S R K Y L A S Q T F

1681 ACGGCGAGCTACCCGTCCCTTAAGGCTACCGCGCGCCTCGCTTCCGTGGCACTCTGGTTT

561 T A S Y P S L K A T A R L A S V A L W F

1741 CTCGTCTTCGGCTGCAAGTTCACCGAGTCGTACTTCTTCCTCACGCTATCGTTCCAGGGT

581 L V F G C K F T E S Y F F L T L S F Q G

1801 CCGATCCGCGTTATGGTGGGTATGAAAATCCAGAATTGCGGCGATAAATATTTCGGCAAT

601 P I R V M V G M K I Q N C G D K Y F G N

1861 GCTCTCTGTCGGAATCAGGCCGCGTTCGCTCTCACGATCATGTACATCATGGACCTGGTG

621 A L C R N Q A A F A L T I M Y I M D L V

1921 CTGTTCTTCCTGGATACGTTCTTGTGGTGGATTATCTGGAACACGGTGGCCAGCATCGCG

641 L F F L D T F L W W I I W N T V A S I A

1981 CGCTCGTTCTACCTTGGATTATCGATCTGGACCCCATGGAAAGACATCTACACTCGTTTG

661 R S F Y L G L S I W T P W K D I Y T R L

2041 CCCAAGCGCATCTATGCGAAGTTGCTCGCTACTGCCGATATGGAAGCGAAGTATAAGCCC

681 P K R I Y A K L L A T A D M E A K Y K P

2101 AAGGTCTTGGTCTCGCAAATCTGGAACGCGATCATTATTTCCATGTACCGCGAGCACTTG

701 K V L V S Q I W N A I I I S M Y R E H L

2161 CTCTCCATTGACCATGTCCAGAAGTTGCTATATCATCAGGTAGACGTTGGCCAAGACGGC

721 L S I D H V Q K L L Y H Q V D V G Q D G

2221 CGCCGCAGCCTGCGTGCTCCCCCGTTCTTTATTTCACAGAGCGACAAGGGGTTCAAGGGC

741 R R S L R A P P F F I S Q S D K G F K G

2281 GAGTTCTTCACGCCCGGAAGCGAGGCGGAGCGCCGAATTTCGTTCTTCGCTCAATCACTC

761 E F F T P G S E A E R R I S F F A Q S L

2341 ACAACTGCTGTTCCGGAGCCTCTACCTGTCGATGCTATGCCCACGTTCACCGTCCTGACG

781 T T A V P E P L P V D A M P T F T V L T

2401 CCTCATTATAGCGAGAAGATCCTTCTTTCATTGAGAGAGATCATTCGCGAAGAGGATCAG

801 P H Y S E K I L L S L R E I I R E E D Q

2461 AATACCCGAGTTACTCTCTTGGAATACCTCAAACAGCTGCACCCCGTCGAATGGGACAAT

821 N T R V T L L E Y L K Q L H P V E W D N

2521 TTCGTCAAGGATACCAAGATCCTCGCCGAGGAGTCAACCATGTACAACGGCCCGAACCCC

841 F V K D T K I L A E E S T M Y N G P N P

2581 TTCGGTTCCGACGAGAAGGGTCAGTCAAAGACCGATGATCTGCCTTTCTATTGCATTGGT

861 F G S D E K G Q S K T D D L P F Y C I G

2641 TTCAAGAGTGCCGCCCCCGAATTCACTCTGCGGACACGTATTTGGGCGTCTCTCCGTGCA

881 F K S A A P E F T L R T R I W A S L R A

2701 CAGACCCTCTACCGTACCGTGTCTGGCATGATGAACTACTCTAAAGCGATTAAGCTACTC

901 Q T L Y R T V S G M M N Y S K A I K L L

2761 TACCGTGTCGAGAACCCCGAGGTCGTTCAGCTCTTCGGCGGCAACACAGACAAGCTGGAG

921 Y R V E N P E V V Q L F G G N T D K L E

2821 CGCGAACTGGAACGCATGGCTCGTCGCAAATTCAAATTCGTCGTTTCGATGCAGCGTTAC

941 R E L E R M A R R K F K F V V S M Q R Y

2881 TCCAAGTTCAACCGCGAGGAGCAAGAGAACGCCGAGTTTCTCCTTCGCGCTTATCCTGAT

961 S K F N R E E Q E N A E F L L R A Y P D

2941 CTCCAGATCGCCTACCTTGAGGAGGAGCCCGCACGCAAGGAAGGTGGCGAAGTCCGACTA

981 L Q I A Y L E E E P A R K E G G E V R L

3001 TTCTCTGCGCTCATCGATGGTCATTCCGAGTTTATCCCTGAGACGGGACGTCGTCGCCCC

1001 F S A L I D G H S E F I P E T G R R R P

3061 AAGTTCCGCATTGAGCTCCCTGGTAACCCAATTCTCGGCGACGGCAAGTCGGACAACCAG

1021 K F R I E L P G N P I L G D G K S D N Q

3121 AACCACGCGATCATCTTCTATCGTGGCGAATACCTGCAGCTCATCGATGCCAACCAGGAT

1041 N H A I I F Y R G E Y L Q L I D A N Q D

3181 AACTACCTGGAGGAGTGTCTGAAGATCCGCAACGTGCTTGGCGAGTTCGAAGAGTACGCC

1061 N Y L E E C L K I R N V L G E F E E Y A

3241 GTCTCCAGCCAGAGCCCTTACGCTCAGTGGGGTCACAAGGAATTCAAGAAGAACCCCGTC

1081 V S S Q S P Y A Q W G H K E F K K N P V

3301 GCTATTGTTGGTGCGCGCGAGTACATCTTCTCCGAGAACATCGGTATTCTGGGTGATTTG

1101 A I V G A R E Y I F S E N I G I L G D L

3361 GCGGCTGGCAAGGAACAGACATTCGGTACGCTCACGGCGCGCTCGCTGGCATGGATTGGG

1121 A A G K E Q T F G T L T A R S L A W I G

3421 GGCAAGCTTCACTATGGCCACCCCGATTTCCTCAACGCGATCTACATGAACACGCGCGGT

1141 G K L H Y G H P D F L N A I Y M N T R G

3481 GGTGTCTCCAAGGCGCAGAAGGGTCTGCACTTGAACGAGGATATCTATGCTGGTATGACC

1161 G V S K A Q K G L H L N E D I Y A G M T

3541 GCGTTTGGCCGCGGTGGACGAATCAAACATACGGAGTACTATCAGTGCGGAAAAGGTCGC

1181 A F G R G G R I K H T E Y Y Q C G K G R

3601 GACCTTGGGTTCGGCACCATTCTGAACTTCCAGACGAAGATCGGTACCGGTATGGGCGAA

1201 D L G F G T I L N F Q T K I G T G M G E

3661 CAGATGCTCAGTCGCGAGTACTACTACCTCGGGACTCAGCTCCCTATCGACCGCTTCCTC

1221 Q M L S R E Y Y Y L G T Q L P I D R F L

3721 ACGTTCTACTACGGCCACCCCGGTTTCCACATCCATAACATGCTCGTCATCCTCTCGGTG

1241 T F Y Y G H P G F H I H N M L V I L S V

3781 CAGATCTTCATCACGACAATGGTGTACTTGGGAACGCTCAAGGGCCAACTCACATTGTGC

1261 Q I F I T T M V Y L G T L K G Q L T L C

3841 CAGTACACTTCAAATGGTGTTTCTCTCGGTCCTACAGGATGCTATAACCTCCAACCTGTG

1281 Q Y T S N G V S L G P T G C Y N L Q P V

3901 TTCCAGTGGATCAATCGCTGTATCATCAGTATCTTTTTAGTGTTCCTGATCGCGTACCTG

1301 F Q W I N R C I I S I F L V F L I A Y L

3961 CCACTTTTCTTGCAAGAACTGGTCGAGCGCGGAACTATAAAAGCCATCCTCCGTCTGACG

1321 P L F L Q E L V E R G T I K A I L R L T

4021 AAGCACTTCAGCTCAGCATCGCCTGCCTTCGAGGTCTTTTCAACGCGTATCTCTTCCCAT

1341 K H F S S A S P A F E V F S T R I S S H

4081 TCCATCGCCACCAATTTAACGTTTGGTGGCGCTCGGTACATCGCGACAGGCCGTGGTTTT

1361 S I A T N L T F G G A R Y I A T G R G F

4141 GCTACTACGAGGATCTCCTTCAGCATTCTCTACTCACGTTTCGCTGGCCCTAGTATATAT

1381 A T T R I S F S I L Y S R F A G P S I Y

4201 CTTGGCATGAGGACGCTCGTGATGCTACTCTATGTCACACTGACGTTGTGGACCGGTTGG

1401 L G M R T L V M L L Y V T L T L W T G W

4261 ATTATCTACTTCTGGTTCTCCACCCTGTCTCTTTGTATCTCGCCATTCTTGTTCAACCCG

1421 I I Y F W F S T L S L C I S P F L F N P

4321 CATCAATTCTCGTTCGCCGATTTCATTGTGGACTATAGGGAGTTCCTGCGTTGGATGAAT

1441 H Q F S F A D F I V D Y R E F L R W M N

4381 CGCGGTAACTCGCGAGTGCACCAGAACTCGTGGATCGGTTACTGCCGTCTTTCACGTACC

1461 R G N S R V H Q N S W I G Y C R L S R T

4441 ATGATCACTGGGTACAAGAAGAAGAAACTCGGACACCCTTCAGAGAAGCTCTCGGGCGAC

1481 M I T G Y K K K K L G H P S E K L S G D

4501 GTTCCCCGGGCAGGTTGGCGCGCTGTGATGTTTTCGGAGATCATCTTCCCCACCGTTATG

1501 V P R A G W R A V M F S E I I F P T V M

4561 GCTGTCCTGATGGTGGTTGCCTACATCTTTGTCAAGTCTTTCCCCGTCGACGCGAGAAAA

1521 A V L M V V A Y I F V K S F P V D A R K

4621 GAGCCACCAAACGCTCTTATTCGTATCGCGATTGTGTCCATCGGACCAATTTTGTGGAAT

1541 E P P N A L I R I A I V S I G P I L W N

4681 GCGGCTATCCTCTTTTTACTATTCCTGGTATCCTTGTTCCTGGGCCCACTGCTGGACACG

1561 A A I L F L L F L V S L F L G P L L D T

4741 CCCTACCCGAAGTTCGGTTCGTTCATGGCCTTCCTTGCTCACTCGTTGGGTGTCATCGGC

1581 P Y P K F G S F M A F L A H S L G V I G

4801 ACGATTGGTTTCTTTGAGTTCCTGTGGTTCCTTGAGTTATGGAACGTTCCTCACGCAGTG

1601 T I G F F E F L W F L E L W N V P H A V

4861 CTGGGTCTCATCGCCGTCATCTACATTCAACGAGCGATCAACAAGGTGCTCATTGCGGTC

1621 L G L I A V I Y I Q R A I N K V L I A V

4921 TTCCTGTCTCGGGAATTCAAGCACGACGAGACGAACCGCGCCTGGTGGACAGGAAAATGG

1641 F L S R E F K H D E T N R A W W T G K W

4981 TACGGTCGCGGTCTGGGCTCGCACGCTATGTCCCAACCAGCGCGCGAGTTCATCGTGAAG

1661 Y G R G L G S H A M S Q P A R E F I V K

5041 ATCATCGAGCTTTCGCTCTGGAGTTCAGATTTCCTCACTGGTCACTTCCTGCTGTTCATT

1681 I I E L S L W S S D F L T G H F L L F I

5101 TTGACCCCACCAATCCTCATACCTTACATTGATCGCGCTCATTCCATGATGCTGTTCTGG

1701 L T P P I L I P Y I D R A H S M M L F W

5161 TTACGTCCTTCGAAACAAATTCGTGCACCTCTGTACTCTATCAAACAGAAGCGGCAGCGC

1721 L R P S K Q I R A P L Y S I K Q K R Q R

5221 CGCTGGATCGTCATCAAATACGGCACCGTCTACGTCCTATCCATTGCTTGCTTTGCTGCC

1741 R W I V I K Y G T V Y V L S I A C F A A

5281 CTGATTGCTCTTCCTCTCGTATTACGAGACGAGCTGCACTTCACGTGCGGCATCTGCCAG

1761 L I A L P L V L R D E L H F T C G I C Q

5341 ACGATCTAG

1781 T I *

(b)

1 ATGGCTGCCCGACCCGGCTATGGTCTACAAAACCCGTTCGCTGGTGGGACTTCCCCATAC

1 M A A R P G Y G L Q N P F A G G T S P Y

61 GGTCCACCTTTGCGTCAGTATTCCAACGATTCCGACCCCGGCGATCCCTATAGTAGTCGT

21 G P P L R Q Y S N D S D P G D P Y S S R

121 AATGCCAGTAGTGTCCCTTTGACACAGGCTGGATACGATGGTCAATCCACTCTATCTACA

41 N A S S V P L T Q A G Y D G Q S T L S T

181 GCCAGCACCGACGATGTGAACGAATACGACCGCCATTATAGCGCATCTGTGGACTCACAC

61 A S T D D V N E Y D R H Y S A S V D S H

241 ATGAGTGGTAGCTCAAGCGCACACGCTCCTTACAACCCGTTCTCTGATATACCTGGCTCC

81 M S G S S S A H A P Y N P F S D I P G S

301 CATGAGCCGTATCCTGCTTGGTCGGCAGAGCGCCAGATACCCATGTCGACAGAAGAGATA

101 H E P Y P A W S A E R Q I P M S T E E I

361 GAAGATATATTTCTGGATCTCGCGCAGAAATTTGGTTTCCAGAGGGATTCCATGCGGAAC

121 E D I F L D L A Q K F G F Q R D S M R N

421 ATGTTCGACTTCCTCATGCATCTCCTCGATTCCCGAGCTTCGCGCATGACACCGAACCAA

141 M F D F L M H L L D S R A S R M T P N Q

481 GCTCTCCTCACTGTCCATGCTGATTATATTGGCGGTCAACACGCGAACTACCGCAAGTGG

161 A L L T V H A D Y I G G Q H A N Y R K W

541 TACTTTGCAGCGCAACTCAACCTAGACGATGCAGTGGGACAGTCACAAAACCCCGGCCTT

181 Y F A A Q L N L D D A V G Q S Q N P G L

601 CAGCGCCTTCGTAGCGTCAAAGGCAAACTTAGCACAGCCAGCACGAAGTCACTTGATAGC

201 Q R L R S V K G K L S T A S T K S L D S

661 GCACTAAACAGATGGCGGAACGCCATGAACAACATGAGCCAGTATGACCGCCTGCGCCAG

221 A L N R W R N A M N N M S Q Y D R L R Q

721 GTAGCACTTTACCTTCTGTGCTGGGCTGAAGCAGGCAACGTACGTTTTACCCCCGAATGT

241 V A L Y L L C W A E A G N V R F T P E C

781 CTGTGTTTCATCTTCAAGTGCGCAGACGACTACTACCGGAGTCCGGAGTGCCAGAACAGA

261 L C F I F K C A D D Y Y R S P E C Q N R

841 GTGGATCCTGTTCCGGAGGGACTGTACCTGGAAGCCGTAATCAAGCCCATCTACAGGTTC

281 V D P V P E G L Y L E A V I K P I Y R F

901 ATGCGTGATCAGGGCTACGAAGTGGTTGAAGGGAAGTTCGTCAGAAGAGAAAAGGATCAC

301 M R D Q G Y E V V E G K F V R R E K D H

961 GATCAGATCATTGGTTACGACGACATTAACCAGCTATTCTGGTACCCCGAAGGAATTGCA

321 D Q I I G Y D D I N Q L F W Y P E G I A

1021 AAGATTGTCCTGCAAGACAATACGCGGCTCATTGACATTCCACCCGCTCAACGTTTCACG

341 K I V L Q D N T R L I D I P P A Q R F T

1081 AAATTCGGGAAGATTGCCTGGAATCGTGTGTTCTTCAAGACCTACTTCGAGAGGCGATCT

361 K F G K I A W N R V F F K T Y F E R R S

1141 ATCGCCCACCTTTTGGTCAACTTCAATCGTATCTGGATCTTACACGTCGCGGTATATTGG

381 I A H L L V N F N R I W I L H V A V Y W

1201 TTCTATACCGCCTTTAATTCACCGAAAGTGTATGCACCGTACAAGAAGGCAGCTCCTTCT

401 F Y T A F N S P K V Y A P Y K K A A P S

1261 GCGGCCATGACTTGGTCTGCGACGGCACTTGGCGGCGCGGTTGCTACCGCAATCATGATT

421 A A M T W S A T A L G G A V A T A I M I

1321 GCTGCGACACTGGCCGAGTTTGCCTACATCCCAACAACATGGAATAACGCGTCCCATCTT

441 A A T L A E F A Y I P T T W N N A S H L

1381 ACGACTAGGCTCATATTCTTAGTCATCGTCCTGGCGCTCACCGGAGGTCCCACCGTGTAT

461 T T R L I F L V I V L A L T G G P T V Y

1441 ATTGCCATGGTTAACGATTCGGCGTCGCACACACCGCTCATCGTCGGAATTGTACAGTTC

481 I A M V N D S A S H T P L I V G I V Q F

1501 TTCGTTTCCGTCGTTGCCACGGTGGCCTTCGGTGTTATTCCATCTGGTCGCATGTTTGGT

501 F V S V V A T V A F G V I P S G R M F G

1561 GATCGGGTCGCGGGCAAGTCGCGCAAATATATGGCATCGCAGACGTTCACAGCGTCCTAC

521 D R V A G K S R K Y M A S Q T F T A S Y

1621 CCTGGTTTGAGCGTTGCCGCCCGTTTGGGGTCTATCTCTCTATGGTCATTGGTCTTCGGC

541 P G L S V A A R L G S I S L W S L V F G

1681 TGCAAGTTTGTGGAATCATACTTCTTCTTGACGTCCTCTTTCAGTAGTCCCATTGCCGTG

561 C K F V E S Y F F L T S S F S S P I A V

1741 ATGGCCCGCACAACGGTGCAAGGTTGTAGCGATAAGTTGTTCGGTACAGCCCTTTGTTCG

581 M A R T T V Q G C S D K L F G T A L C S

1801 AATCAAGTGCCATTCGCGCTCGCAATCATGTACGTGATGGACCTAGTGCTGTTCTTCCTG

601 N Q V P F A L A I M Y V M D L V L F F L

1861 GACACGTACCTCTGGTACATCATCTGGGTGGTGGTGTTTAGTGTCTCCCGCTCCTTCTAT

621 D T Y L W Y I I W V V V F S V S R S F Y

1921 CTCGGTCTGTCGATTTGGACGCCCTGGAAAGAGGTATATACCAGGATGCCGAAGAGGATC

641 L G L S I W T P W K E V Y T R M P K R I

1981 TACGCGAAGCTACTCGCTACTGCGGAGATGGAGATCAAATACAAACCGAAGGTTCTTGTT

661 Y A K L L A T A E M E I K Y K P K V L V

2041 TCTCAAGTATGGAATGCCATCATCATTTCCATGTACCGCGAACATCTTTTATCTATCCAC

681 S Q V W N A I I I S M Y R E H L L S I H

2101 CATGTGCAACGTCTCCTATATCATCAAGTTGATGGACCAGACGGTCGCCGTGTCCTTCGA

701 H V Q R L L Y H Q V D G P D G R R V L R

2161 GCTCCTCCGTTCTTCACTCATAATAACGGCAACGAAAGTGACTTCTTCCCTCCAGGCGGC

721 A P P F F T H N N G N E S D F F P P G G

2221 GAGGCTGAACGTCGTATCTCCTTCTTTGCGTCATCCCTCACCACTGCGCTTCCCGATCCT

741 E A E R R I S F F A S S L T T A L P D P

2281 CTTCCGGTTGATGCCATGCCGACGTTCACGGTTCTAGTACCCCATTACTCCGAGAAGATC

761 L P V D A M P T F T V L V P H Y S E K I

2341 CTGCTGAGCTTGCGTGAGATTATCAGGGAGGAGGATCAGAATACTCGCGTTACCCTCCTC

781 L L S L R E I I R E E D Q N T R V T L L

2401 GAATATCTGAAGCAGCTACACCCTGTTGAATGGGACAATTTCGTGAAAGATACCAAGATT

801 E Y L K Q L H P V E W D N F V K D T K I

2461 TTGGCGGAGGAGTCGGAGACGTCAACGTTTGACGGCACTGGTACTACTAACGAGAAGTCA

821 L A E E S E T S T F D G T G T T N E K S

2521 AACAACCGCACGGATGATCTGCCATTCTACTGCATTGGATTCAAGACTGCCGCACCCGAG

841 N N R T D D L P F Y C I G F K T A A P E

2581 TACACTCTTCGTACCCGCATCTGGGCTTCGCTCCGCGCGCAGACACTCTACCGTACGGTG

861 Y T L R T R I W A S L R A Q T L Y R T V

2641 TCCGGGATGATGAACTATGCCAAGGCCATCAAGCTGTTGTATCGCGTCGAGAACCCGCAG

881 S G M M N Y A K A I K L L Y R V E N P Q

2701 ATTGTGCAGCGGTTCGCCGGGAACACGGACAGACTCGAGCGTGAGCTGGAGCGTATGTCG

901 I V Q R F A G N T D R L E R E L E R M S

2761 AGGCGGAAGTTCAAGTTCACTGTGTCCATGCAGCGGTATGCCAAGTTCAACAAGGAGGAG

921 R R K F K F T V S M Q R Y A K F N K E E

2821 CTTGAGAACGCCGAGTTTCTCCTGCGTGCATACCCGGATCTGCAGATTGCATACCTCGAC

941 L E N A E F L L R A Y P D L Q I A Y L D

2881 GAAGAACCCGGTCCGAAGGGTGGAGACCCGCGATTGTTTTCTGTACTCATTGATGGTCAT

961 E E P G P K G G D P R L F S V L I D G H

2941 TCAGAGGTGGACAGCAAGACTGGGAAGCGGATGCCCAAGTTCCGCATTGAGCTTCCCGGC

981 S E V D S K T G K R M P K F R I E L P G

3001 AATCCCATTCTCGGTGATGGCAAGTCCGACAACCAGAATCATGCGATCATCTTCTACCGT

1001 N P I L G D G K S D N Q N H A I I F Y R

3061 GGCGAATACCTGCAGCTCATCGATGCCAACCAGGATAACTACCTGGAGGAATGCATCAAG

1021 G E Y L Q L I D A N Q D N Y L E E C I K

3121 ATTCGGAGCATCCTTGGTGAGTTTGAGGAATACACTATCTCCAGTCAAAGCCCGTATGCA

1041 I R S I L G E F E E Y T I S S Q S P Y A

3181 CAATGGGGTCACAAGGAGTTCAAAAGATCCCCTGTGGCGATCGTGGGTACTCGCGAGTAC

1061 Q W G H K E F K R S P V A I V G T R E Y

3241 ATCTTCTCAGAGAACATTGGTGTTCTGGGTGACATTGCAGCAGGTAAGGAACAGACGTTC

1081 I F S E N I G V L G D I A A G K E Q T F

3301 GGTACGATGACTCCGCGCGTTTTGGCTTGGATCGGCGGCAAGCTGCACTACGGTCACCCC

1101 G T M T P R V L A W I G G K L H Y G H P

3361 GATTTCTTGAACGCGGCATTCATGGCCACGCGCGGTGGTGTCTCTAAGGCTCAGAAGGGT

1121 D F L N A A F M A T R G G V S K A Q K G

3421 CTGCATCTTAACGAGGACATTTTCGCTGGTATGAACGCTATCGGCCGTGGTGGGCGCATC

1141 L H L N E D I F A G M N A I G R G G R I

3481 AAACACTCGGAGTACTACCAGTGCGGAAAGGGTCGTGACCTTGGCTTCGGCACCATCCTG

1161 K H S E Y Y Q C G K G R D L G F G T I L

3541 AACTTCCAGACGAAACTGGGGACTGGTATGGGTGAGCAAATGCTTAGCAGAGAGTACTAC

1181 N F Q T K L G T G M G E Q M L S R E Y Y

3601 TATCTGGGTACCCAGTTACCCTTGGACCGTTTCCTCACCTTCTATTATGGACATCCTGGA

1201 Y L G T Q L P L D R F L T F Y Y G H P G

3661 TTCCACATCAACAACATCTTGGTTATCTACTCCATCCAGATCTTCATGGTCACTCTCTTG

1221 F H I N N I L V I Y S I Q I F M V T L L

3721 TATATCGGCACATTGAATAAGGAACTCGCGGTTTGCATTATCAATTCGCAAGGGGACGTT

1241 Y I G T L N K E L A V C I I N S Q G D V

3781 ATTGGTGGTCAGCCCGGTTGTTACAATTTAACCCCCGTCTTCCAGTGGATTAAACGATGC

1261 I G G Q P G C Y N L T P V F Q W I K R C

3841 ATCATATCCATCTTTTTGGTCTTCTTCATCGCTTTCTTGCCACTATTCTTGCAAGAACTG

1281 I I S I F L V F F I A F L P L F L Q E L

3901 CTCGAACGAGGCACAGGAAAGGCATTGATTCGTCTTGGAAAACATTTCCTATCCCTTTCT

1301 L E R G T G K A L I R L G K H F L S L S

3961 CCCATTTTCGAAGTCTTTTCCACCCAGATCTACTCGCAGTCCATCTTGAGCAATCTGACG

1321 P I F E V F S T Q I Y S Q S I L S N L T

4021 TTCGGCGGCGCGCGGTACATCGCCACGGGACGTGGATTTGCAACCACGCGGATATCCTTC

1341 F G G A R Y I A T G R G F A T T R I S F

4081 ACCATACTCTATTCCCGCTTCGCTGGTCCTAGTATCTATATGGGCATGCGCAACGTTCTT

1361 T I L Y S R F A G P S I Y M G M R N V L

4141 CTACTCTTGTATGCCACTATGGCCATCTGGACACCGTACCTGATCTACTTCTGGTTCTCG

1381 L L L Y A T M A I W T P Y L I Y F W F S

4201 GTCCTCTCATTGTGTATCGCGCCTTTCGTATTCAACCCGCATCAGTTCTCTTTCCCCGAC

1401 V L S L C I A P F V F N P H Q F S F P D

4261 TTCATCATCGATTATCGGGAGTTCTTGCGTTGGATGTCTCGCGGCAATTCTCGCACAAAG

1421 F I I D Y R E F L R W M S R G N S R T K

4321 GCCAGCAGTTGGTACGGCTATTGTCGTCTCTCGCGAACGATGATCACTGGTTACAAGAAG

1441 A S S W Y G Y C R L S R T M I T G Y K K

4381 AAGAAACTCGGTCATCCTTCGGAGAAGTTATCTGGAGACGTCCCCCGCGCAAGGTGGAAA

1461 K K L G H P S E K L S G D V P R A R W K

4441 GCCGTCATCTTCTCTGAGATCGTGTGGCCGATCTGTCAAGCTACGATATTTGTTGTCGCG

1481 A V I F S E I V W P I C Q A T I F V V A

4501 TATATGTTCGTGAAATCGTTCCCGAATCACCTCGGGCAACAGAATCCCAGTCCATTCATC

```
1501   Y  M  F  V  K  S  F  P  N  H  L  G  Q  Q  N  P  S  P  F  I
4561   CGCATTGCCGTCATTGCTGTTGGGCCCGTCGTATGGAACGCGGCGATCTTGTTGGCTCTG
1521   R  I  A  V  I  A  V  G  P  V  V  W  N  A  A  I  L  L  A  L
4621   TTCTTCATTTCGTTGTTCCTAGGGCCGATGATGGAATCTTGGACGAGATTCGCTTCGGTA
1541   F  F  I  S  L  F  L  G  P  M  M  E  S  W  T  R  F  A  S  V
4681   ATGGCCGCACTTGCTCATTTCCTCGCCCTGTTCGGTCTTATCGCATTTTTCGAATTCTTC
1561   M  A  A  L  A  H  F  L  A  L  F  G  L  I  A  F  F  E  F  F
4741   TGGTTCCTCGAGCTGTGGGATGCGTCACACGCCGTTCTTGGGGCGATCTCCATCATCGCT
1581   W  F  L  E  L  W  D  A  S  H  A  V  L  G  A  I  S  I  I  A
4801   ATCCAGCGCGCCATCCAGAAGATCCTGATAGCTGTCTTCCTGTCGCGGGAGTACAAGCAT
1601   I  Q  R  A  I  Q  K  I  L  I  A  V  F  L  S  R  E  Y  K  H
4861   GACGAGACGAATCGTGCCTGGTGGTCAGGTAAATGGTATGGACGGGGACTAGGCAATTCT
1621   D  E  T  N  R  A  W  W  S  G  K  W  Y  G  R  G  L  G  N  S
4921   GCTTTGTCGCAACCTGCGCGCGAGTATGTGGTGAAGATAGTTGAGATGTCTTTGTGGAGC
1641   A  L  S  Q  P  A  R  E  Y  V  V  K  I  V  E  M  S  L  W  S
4981   TCGGATTTCTTATTGGCACACATATTGTTGATTATCATGACGCCTCCTACCCTCATCCCC
1661   S  D  F  L  L  A  H  I  L  L  I  I  M  T  P  P  T  L  I  P
5041   TTTGTCGACAAGCTGCATTCCACGATGATGTTCTGGTTACGACCATCCAAGCAGATACGA
1681   F  V  D  K  L  H  S  T  M  M  F  W  L  R  P  S  K  Q  I  R
5101   CCGCCACTTTTCTCAACGAAACAAAGACGGCAACGTCGTTGGATCGTGGTGAAATACACC
1701   P  P  L  F  S  T  K  Q  R  R  Q  R  R  W  I  V  V  K  Y  T
5161   TTCGTTTACATTGTAGTAGTGGCATGCCTCGCTGCGCTGATCGTGCTCCCTGCCCTCTTC
1721   F  V  Y  I  V  V  V  A  C  L  A  A  L  I  V  L  P  A  L  F
5221   CGAGATCGCATCACGTTTAACTGTACACTATGTCAGAATATCTAA
1741   R  D  R  I  T  F  N  C  T  L  C  Q  N  I  *
```

Fig. S3: **Biosynthetic gene clusters in the *S. crispa* genome.**

The genes with predicted functions are: core biosynthetic genes (■), additional biosynthetic genes (■), transport-related genes (■), regulatory genes (■) and other genes (■).

contig_000000F - Gene Cluster 1. Type = other. Location: 298010 - 342192 nt.

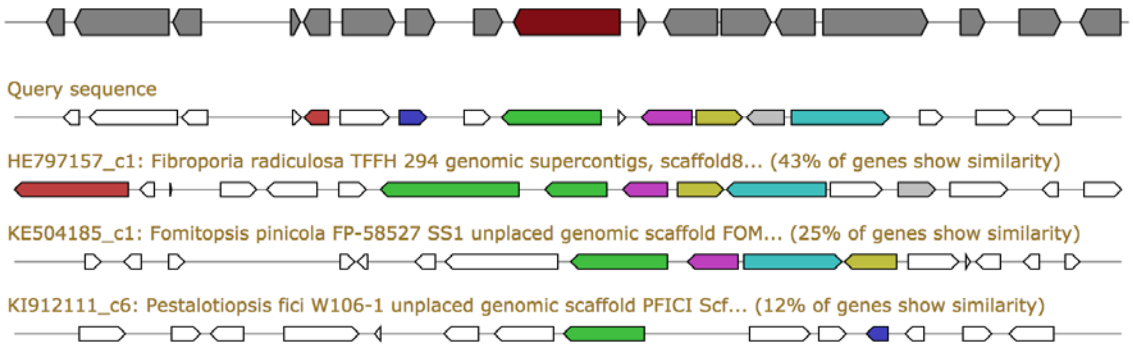

contig_000000F - Gene Cluster 2. Type = other. Location: 2151671 - 2195752 nt.

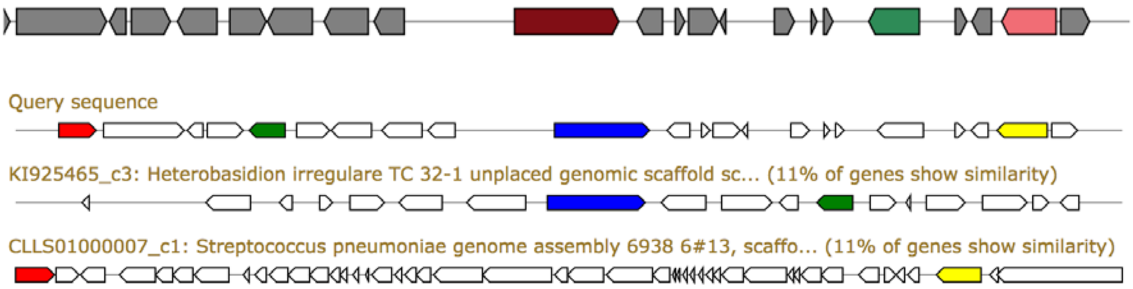

contig_000001F - Gene Cluster 3. Type = terpene. Location: 577100 - 598315 nt.

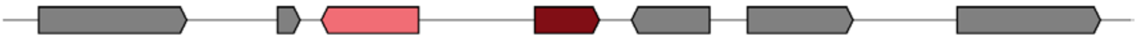

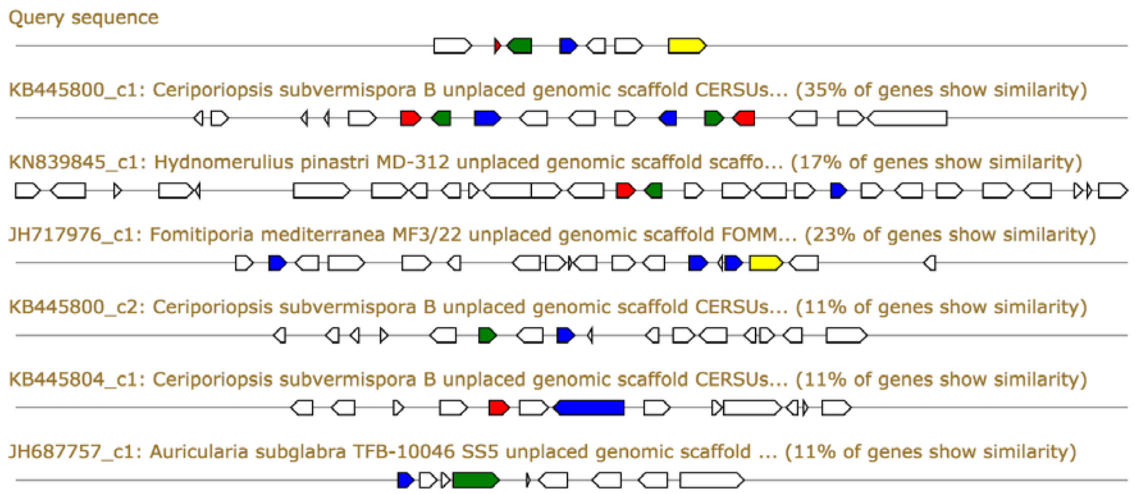

contig_000001F - Gene Cluster 4. Type = other. Location: 1226000 - 1279843 nt.

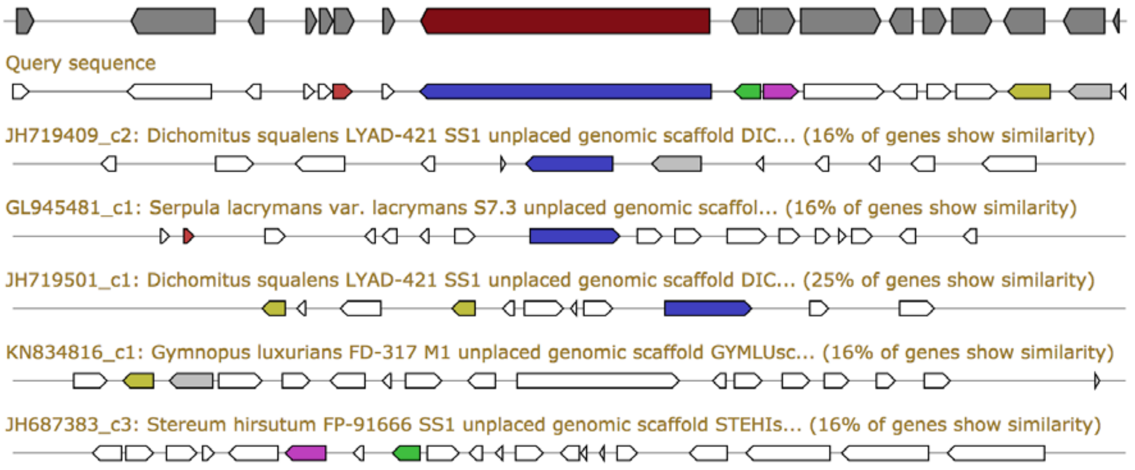

contig_000002F - Gene Cluster 5. Type = terpene. Location: 1420916 - 1442540 nt.

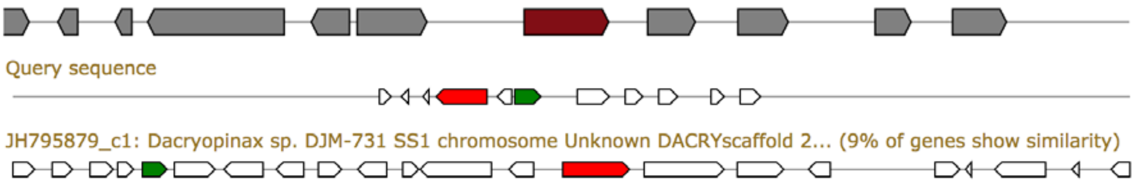

contig_000002F - Gene Cluster 6. Type = t1pks. Location: 1903728 - 1956364 nt.

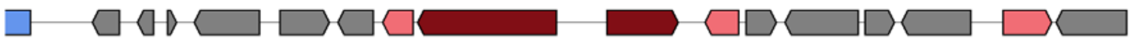

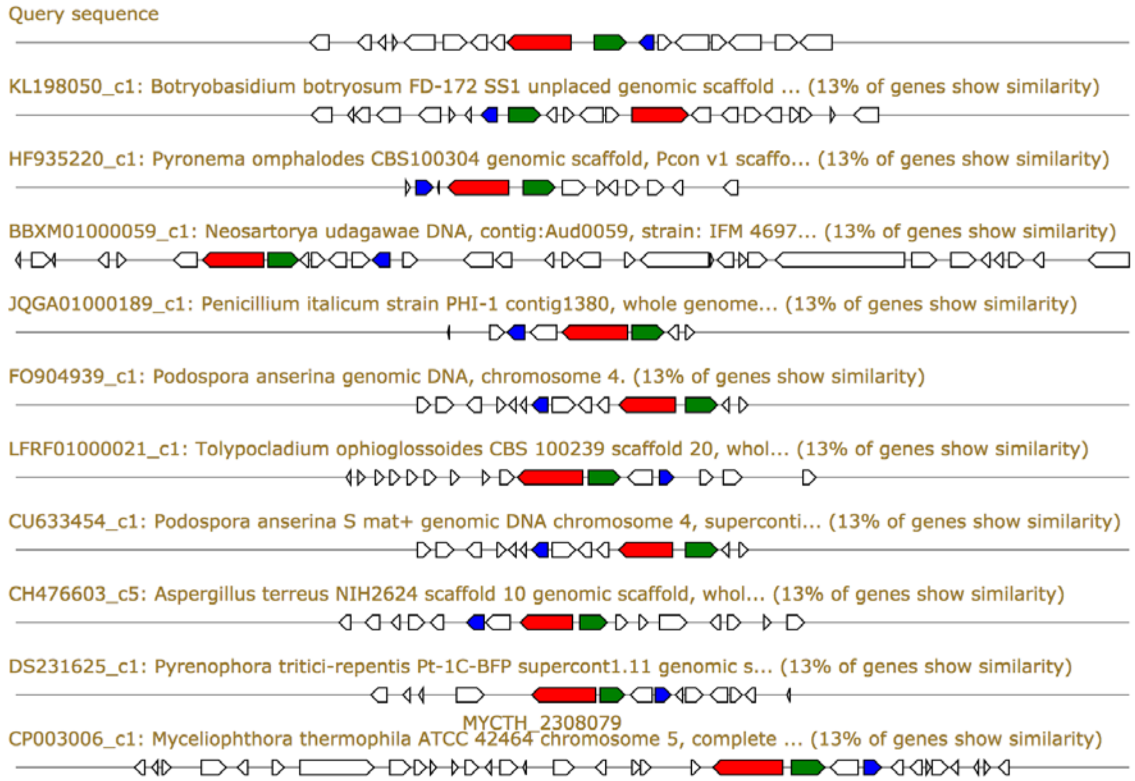

contig_000002F - Gene Cluster 7. Type = terpene. Location: 2394718 - 2415961 nt.

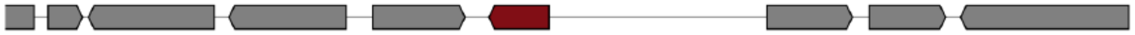

contig_000003F - Gene Cluster 8. Type = terpene. Location: 780278 - 801362 nt.

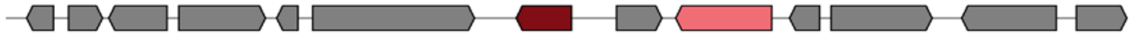

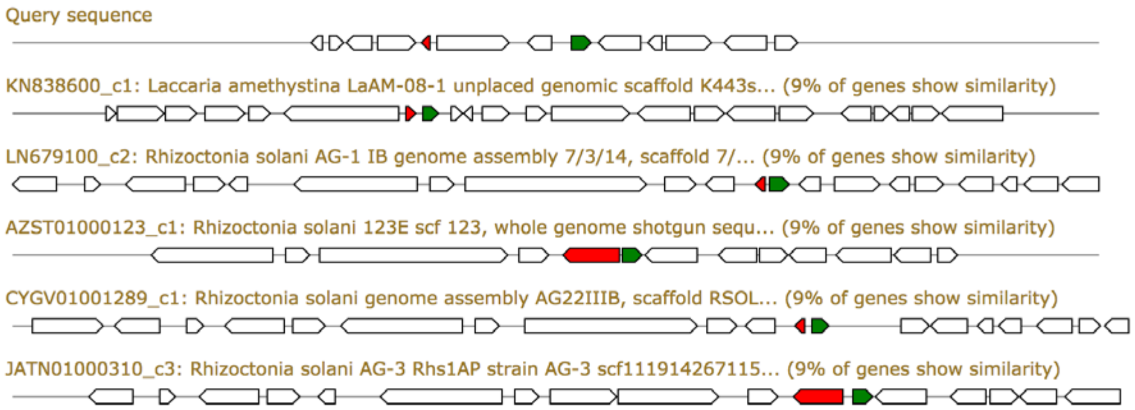

contig_000003F - Gene Cluster 9. Type = terpene. Location: 1471027 - 1501332 nt.

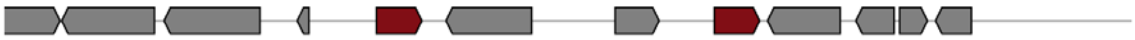

contig_000003F - Gene Cluster 10. Type = terpene. Location: 1523295 - 1544372 nt.

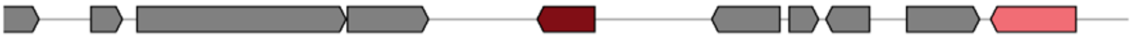

contig_000004F - Gene Cluster 11. Type = other. Location: 2837290 - 2881205 nt.

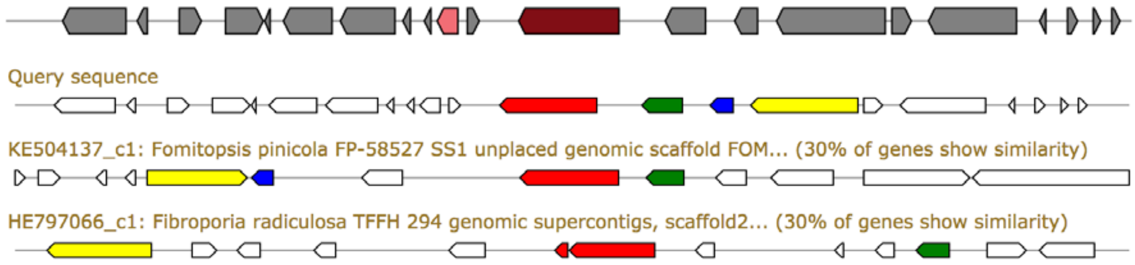

contig_000005F - Gene Cluster 12. Type = indole. Location: 730480 - 752441 nt.

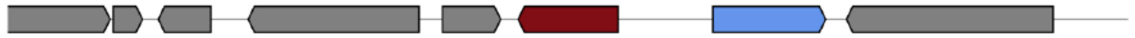

contig_000005F - Gene Cluster 13. Type = terpene. Location: 1217614 - 1239213 nt.

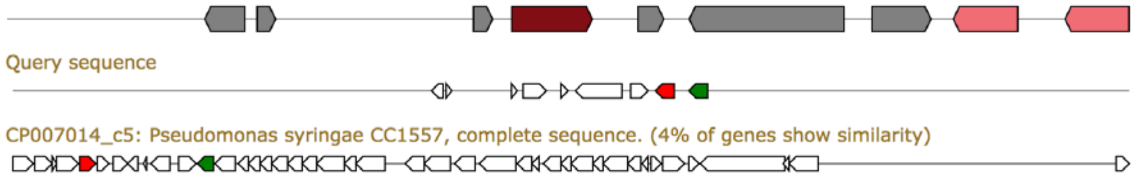

contig_000005F - Gene Cluster 14. Type = other. Location: 1681418 - 1725375 nt.

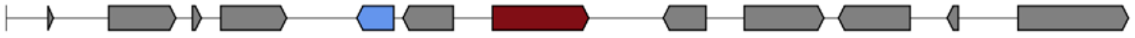

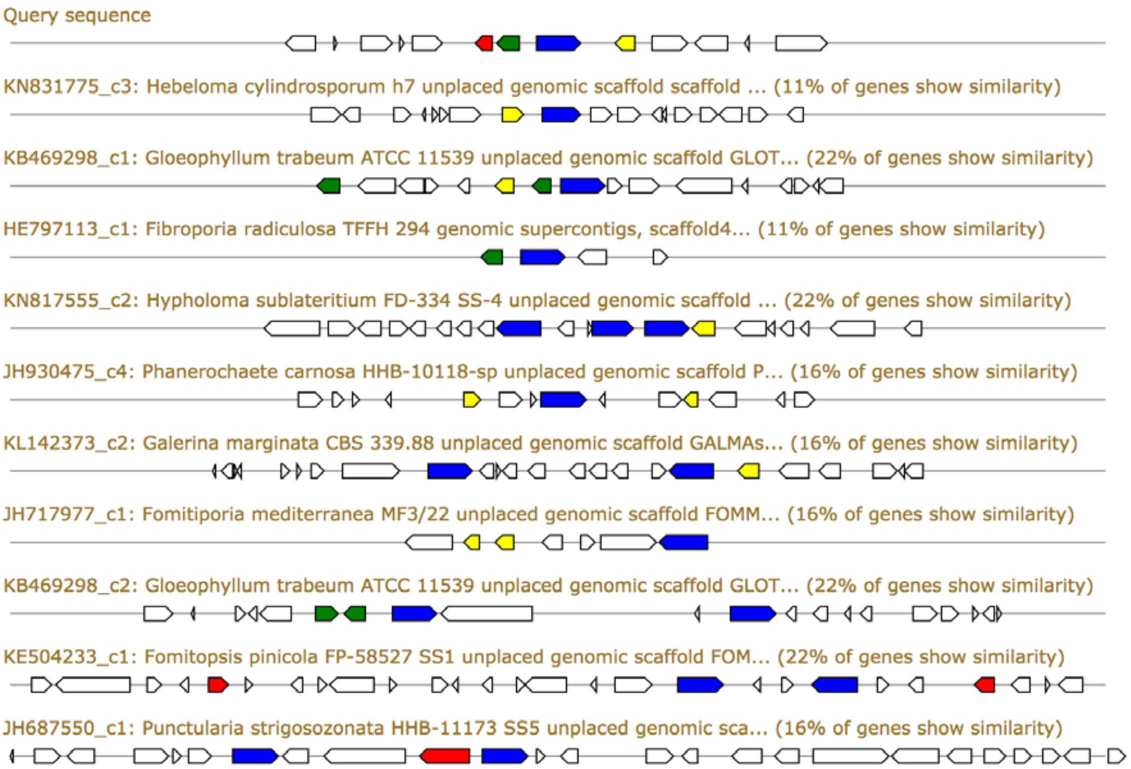

contig_000006F - Gene Cluster 15. Type = terpene. Location: 344484 - 365726 nt.

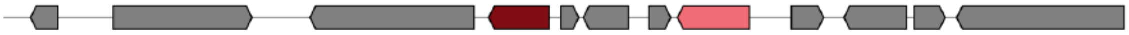

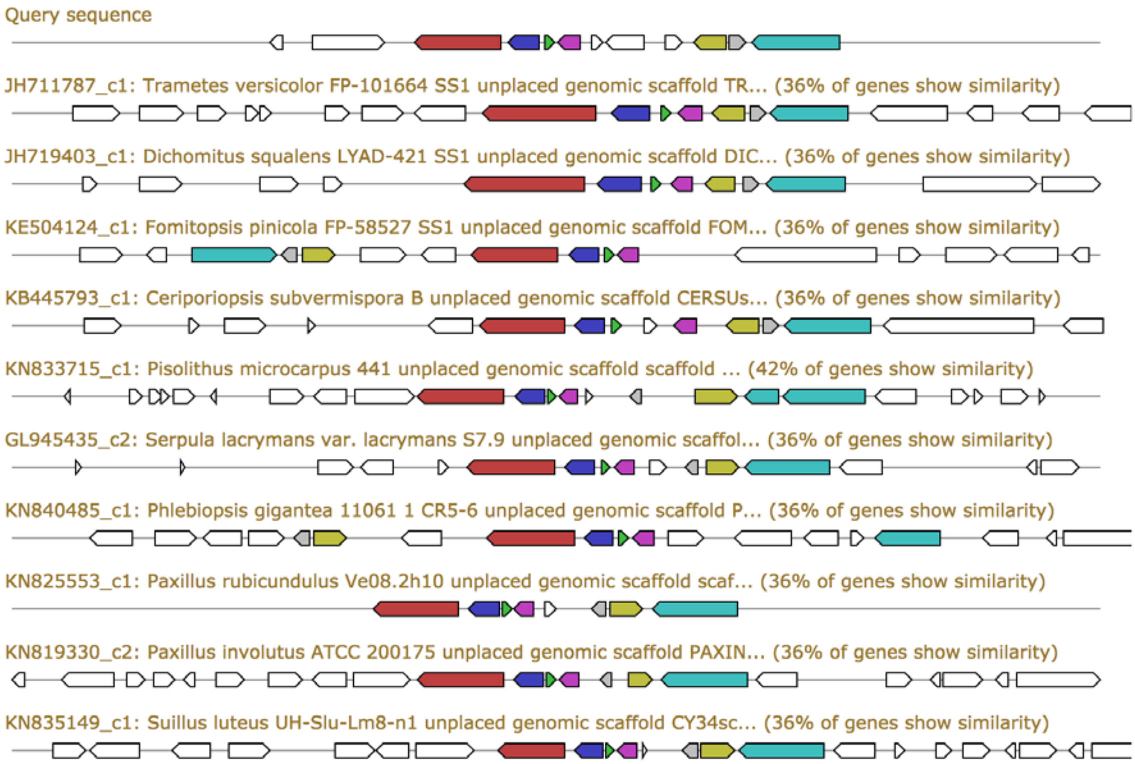

contig_000007F - Gene Cluster 16. Type = terpene. Location: 1818743 - 1839963 nt.

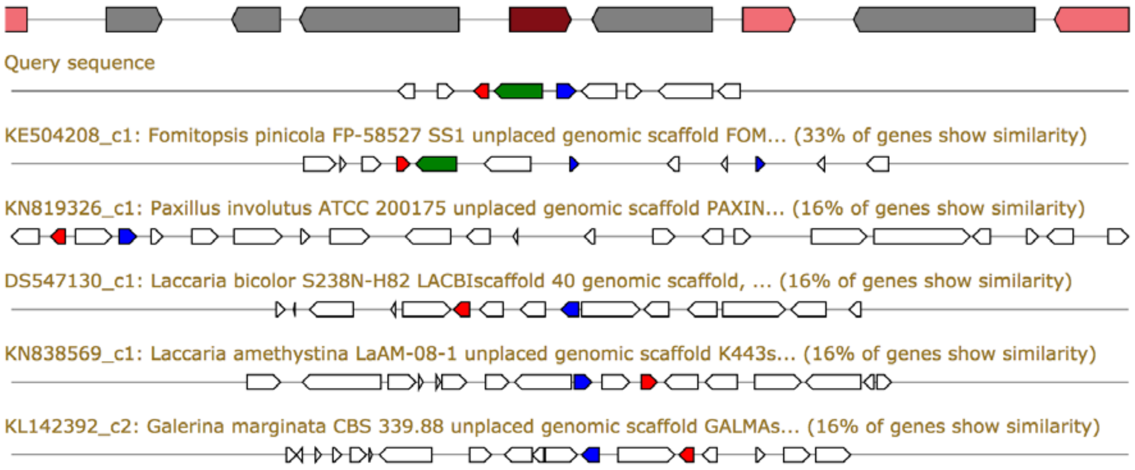

contig_000008F - Gene Cluster 17. Type = terpene. Location: 106419 - 130408 nt.

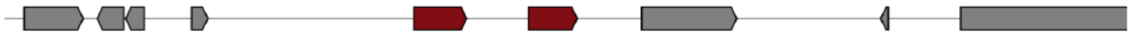

contig_000008F - Gene Cluster 18. Type = other. Location: 882452 - 926420 nt.

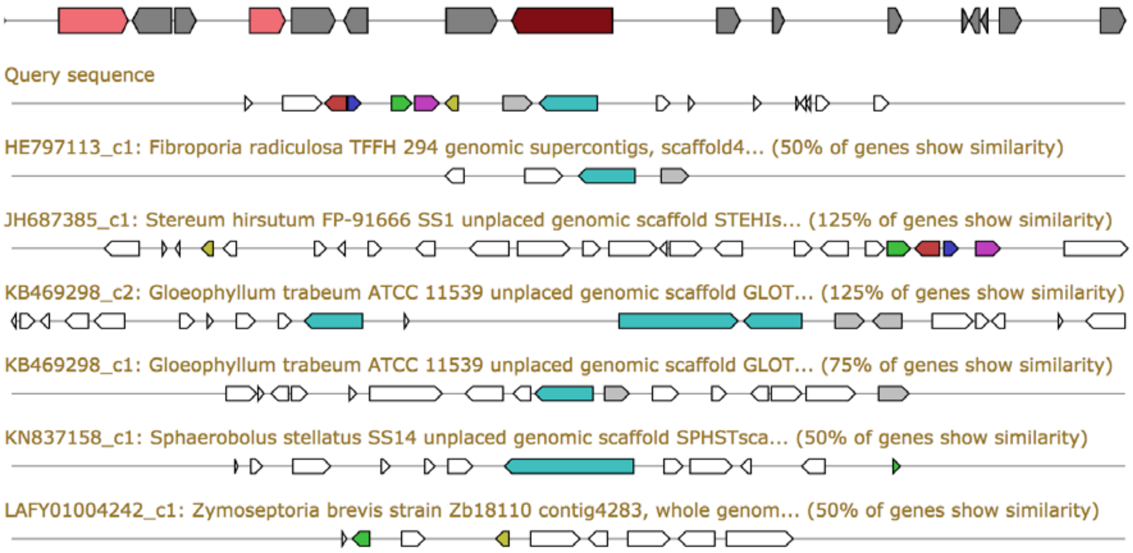

contig_000008F - Gene Cluster 19. Type = terpene. Location: 1136595 - 1159210 nt.

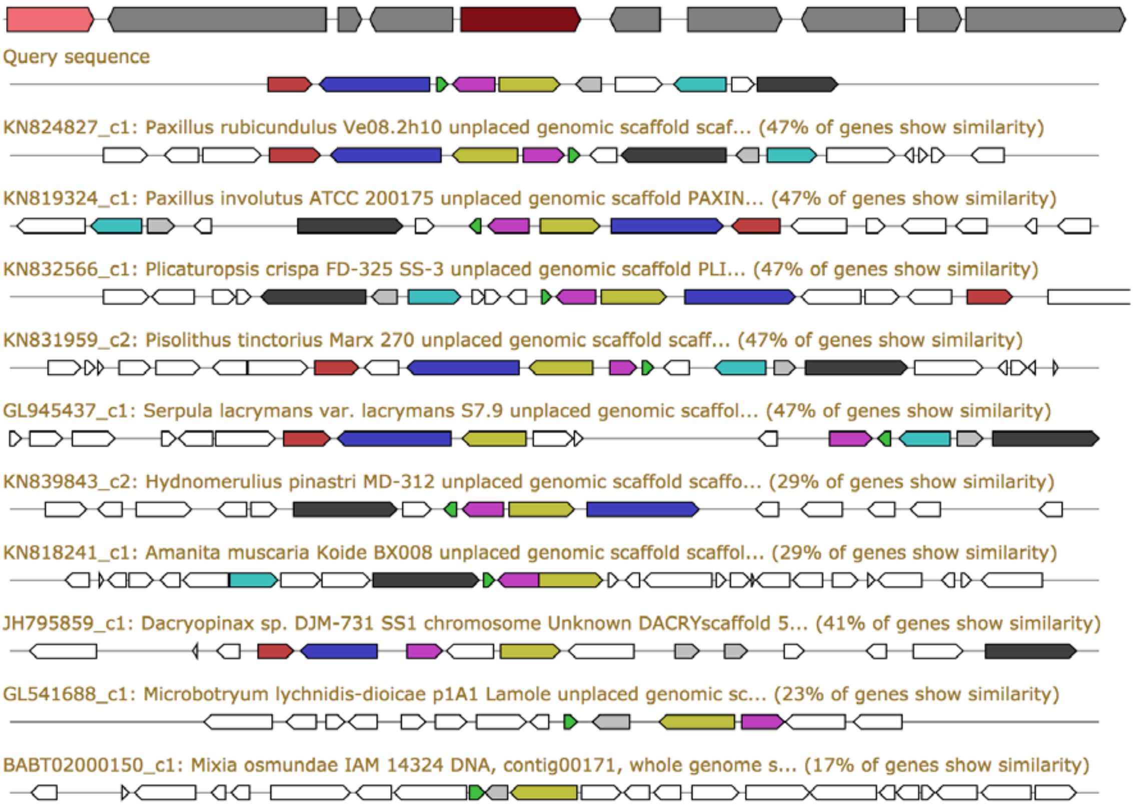

contig_000008F - Gene Cluster 20. Type = terpene. Location: 1472478 - 1494300 nt.

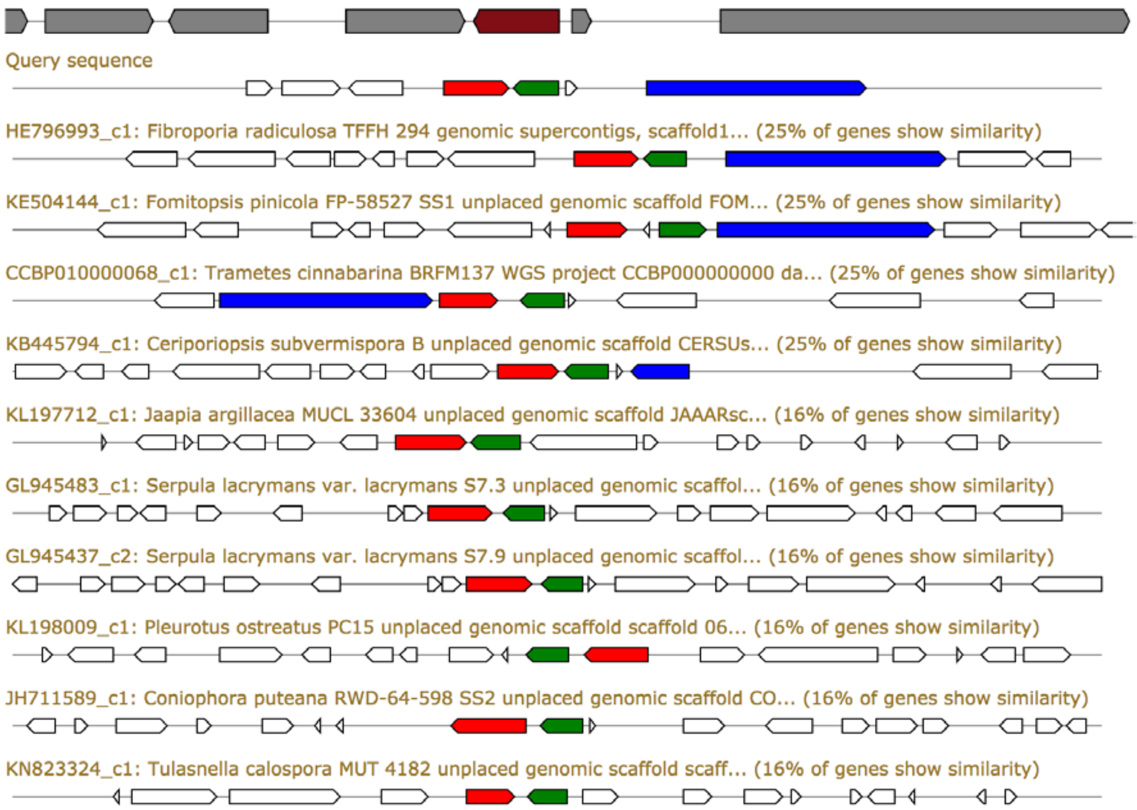

contig_000011F - Gene Cluster 21. Type = other. Location: 351494 - 398797 nt.

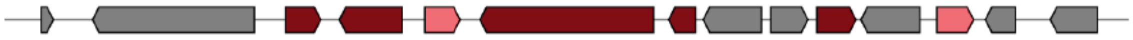

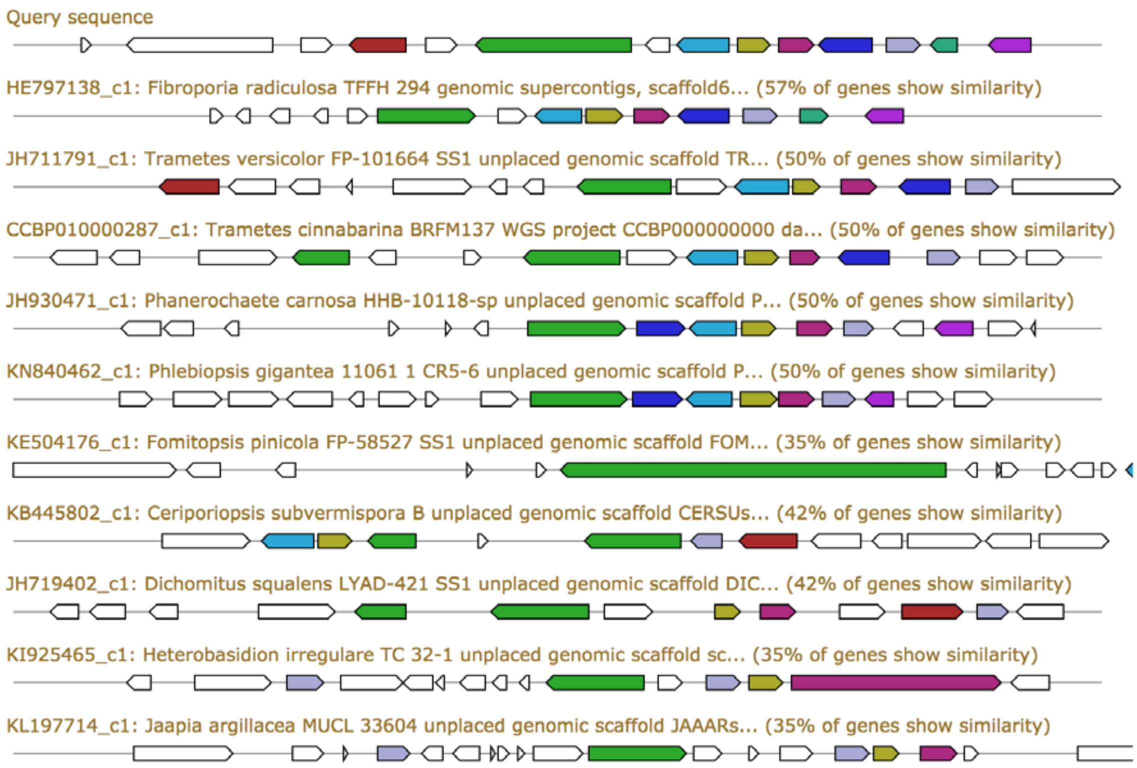

contig_000010F - Gene Cluster 22. Type = t1pks. Location: 885689 - 934146 nt.

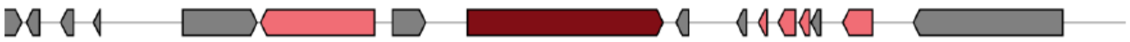

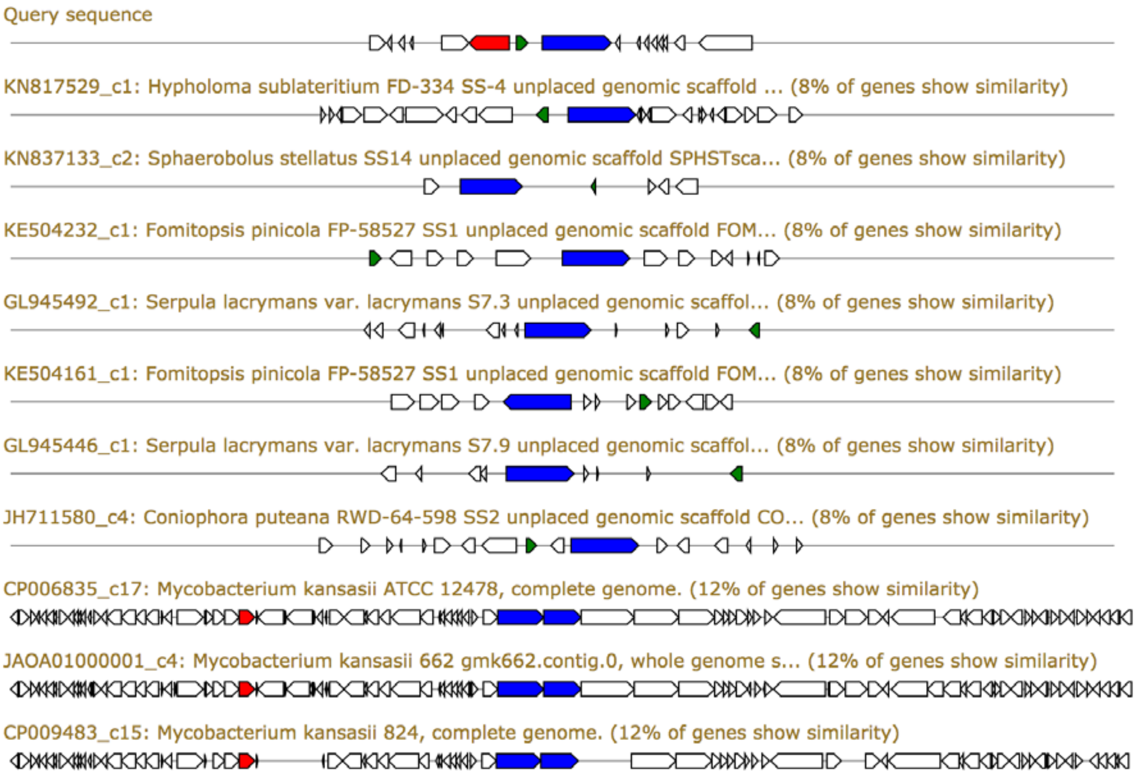

contig_000010F - Gene Cluster 23. Type = indole-nrps. Location: 1041798 - 1097259 nt.

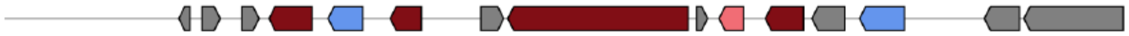

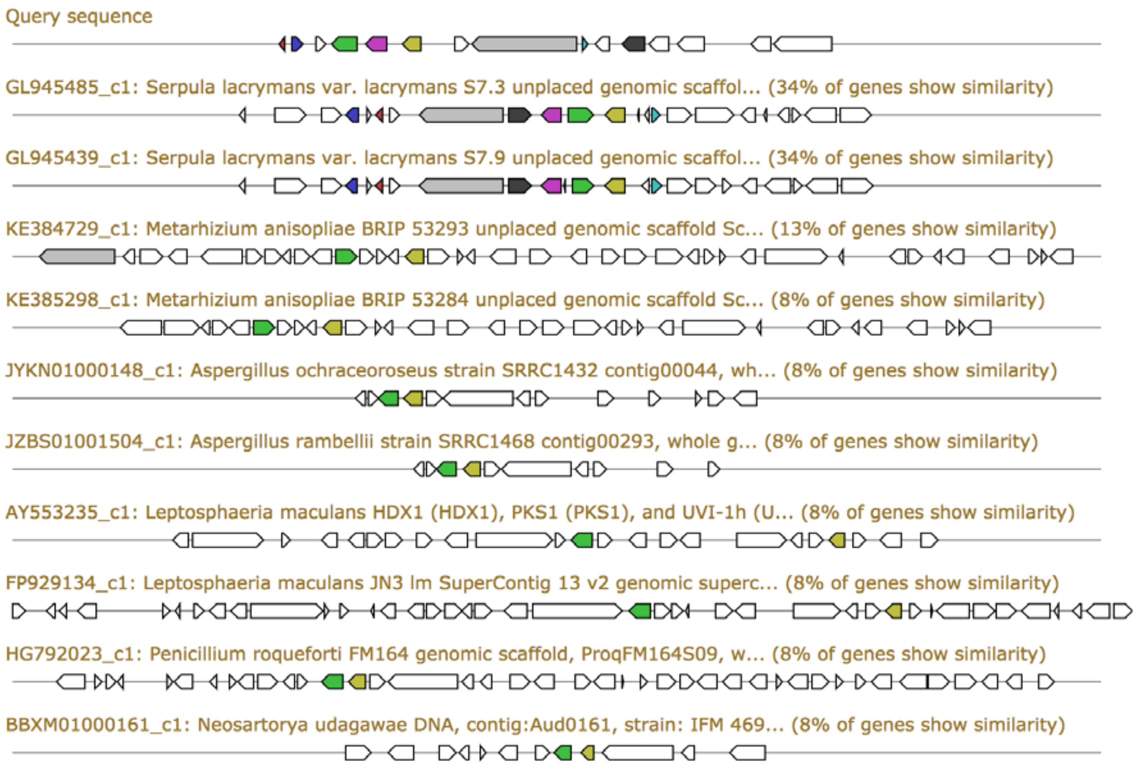

contig_000012F - Gene Cluster 24. Type = terpene. Location: 174101 - 201152 nt.

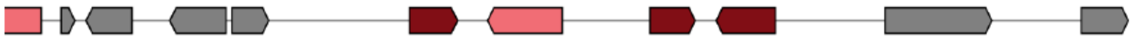

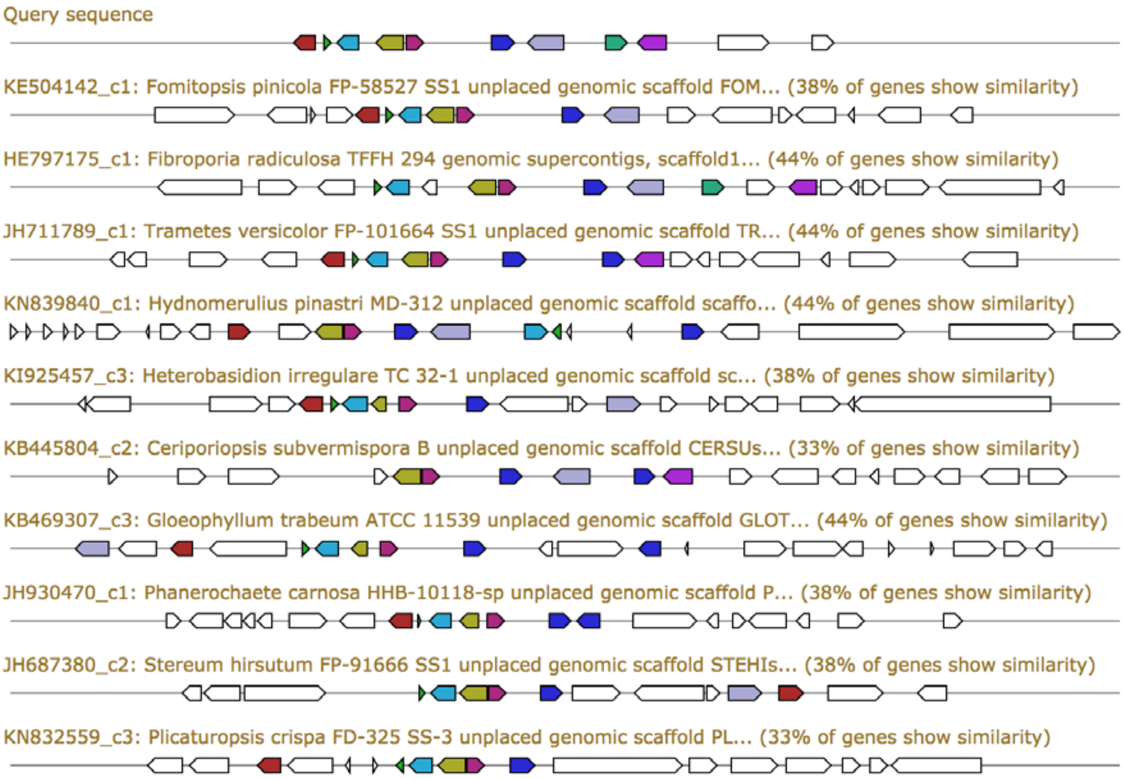

contig_000013F - Gene Cluster 25. Type = terpene. Location: 466945 - 488536 nt.

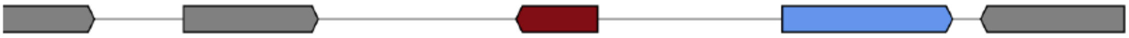

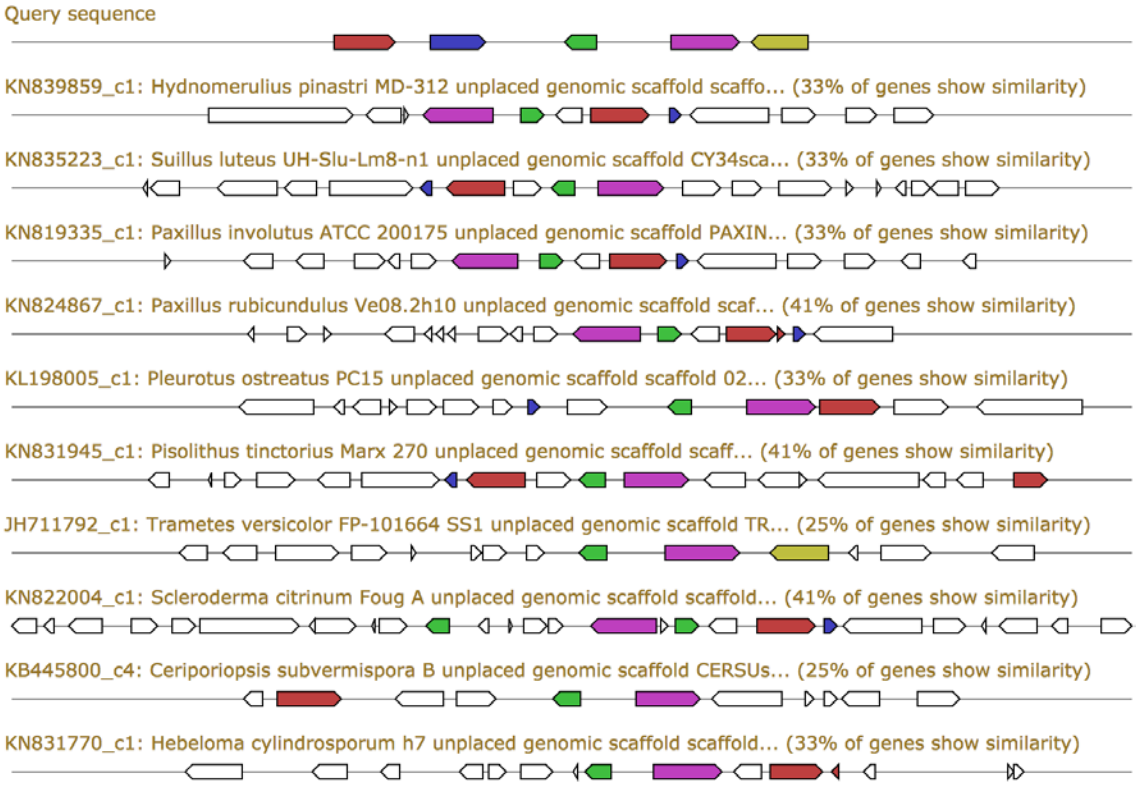

contig_000014F - Gene Cluster 26. Type = terpene. Location: 887958 - 909591 nt.

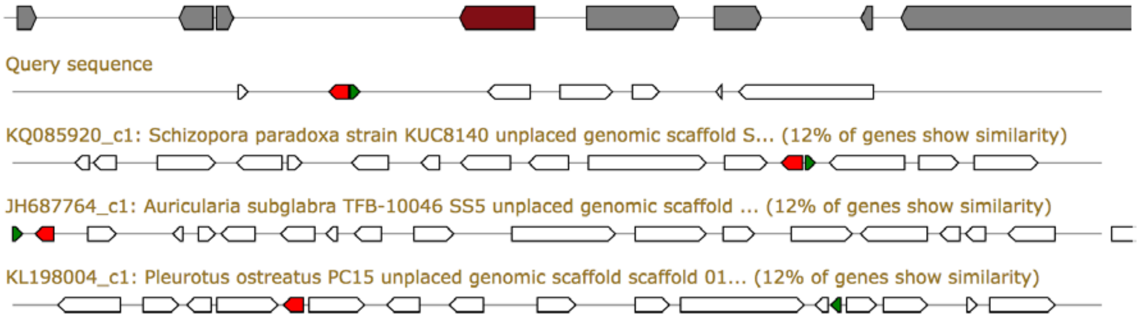

contig_000015F - Gene Cluster 27. Type = t1pks. Location: 462780 - 507320 nt.

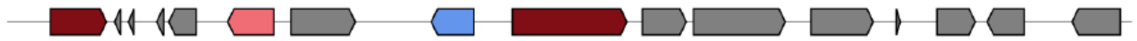

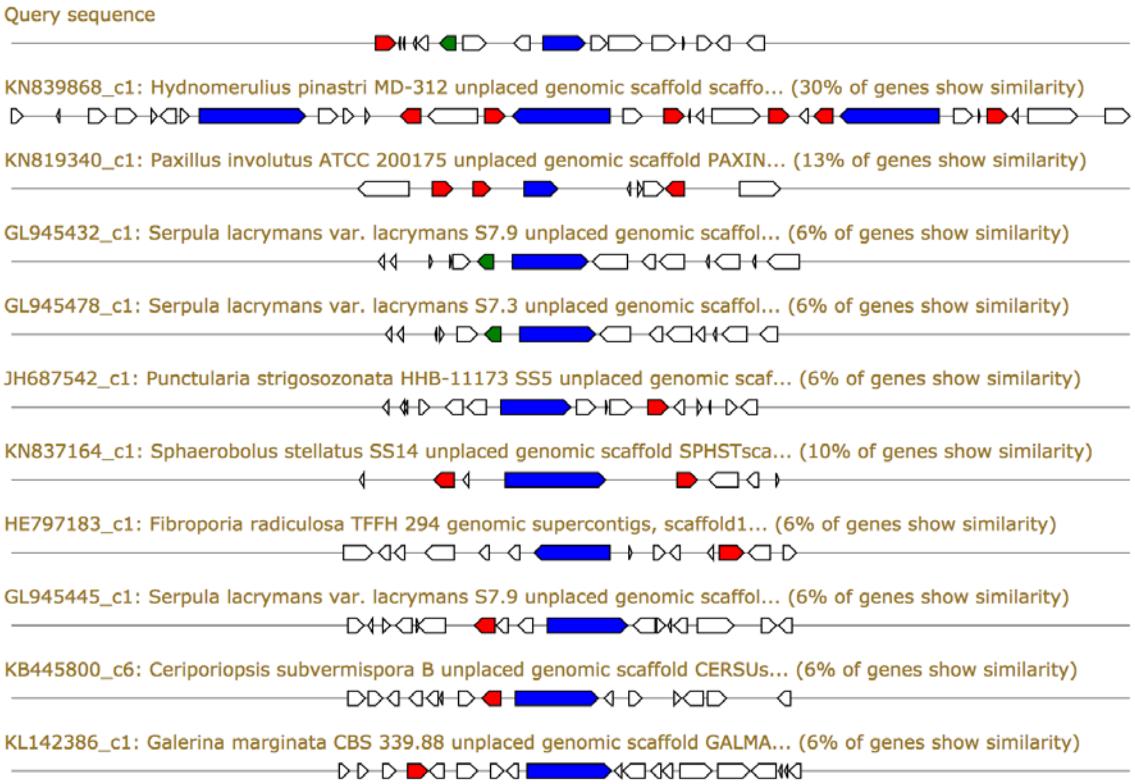

contig_000016F - Gene Cluster 28. Type = other. Location: 119822 - 164105 nt.

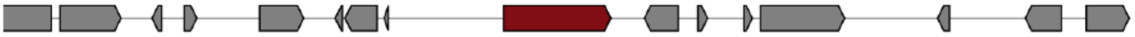

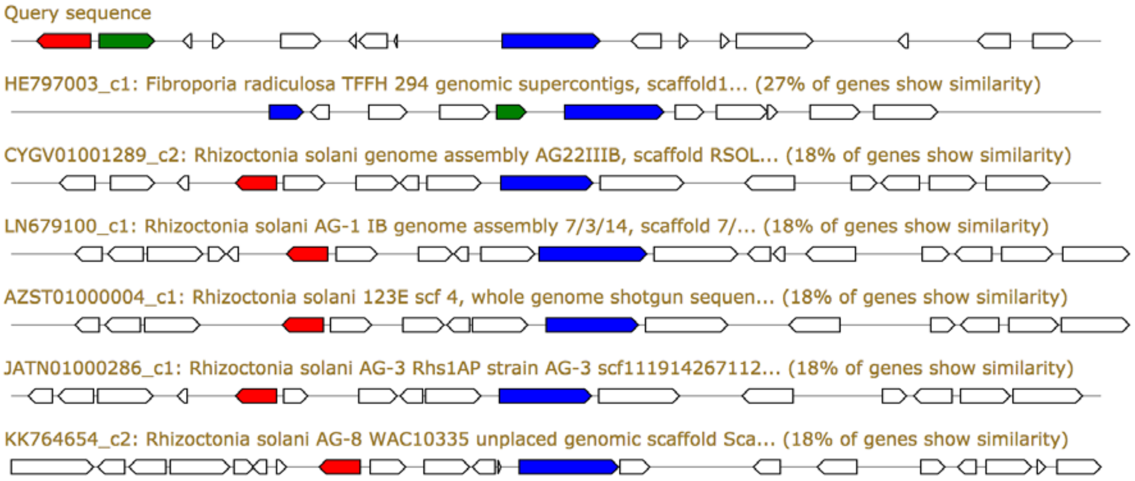

contig_000016F - Gene Cluster 29. Type = t1pks. Location: 567660 - 616617 nt.

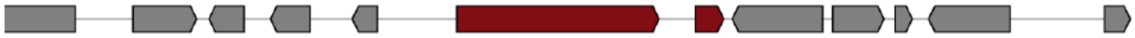

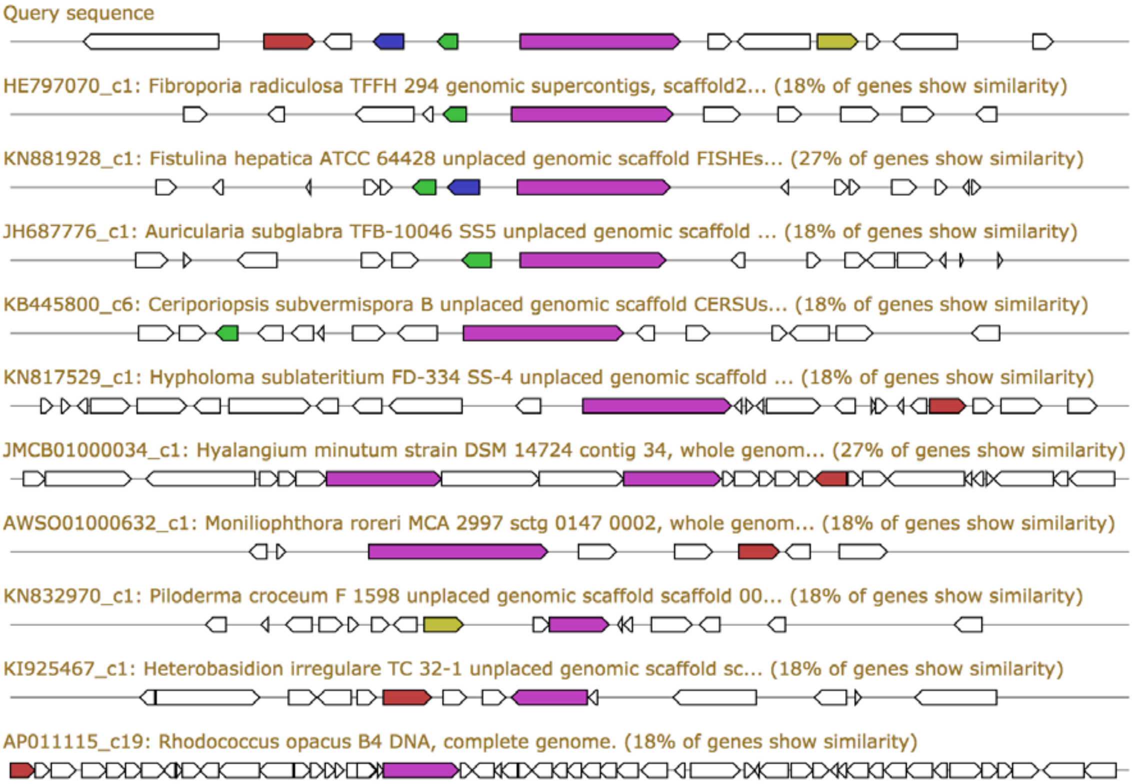

contig_000016F - Gene Cluster 30. Type = t1pks. Location: 678964 - 727930 nt.

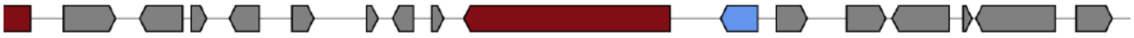

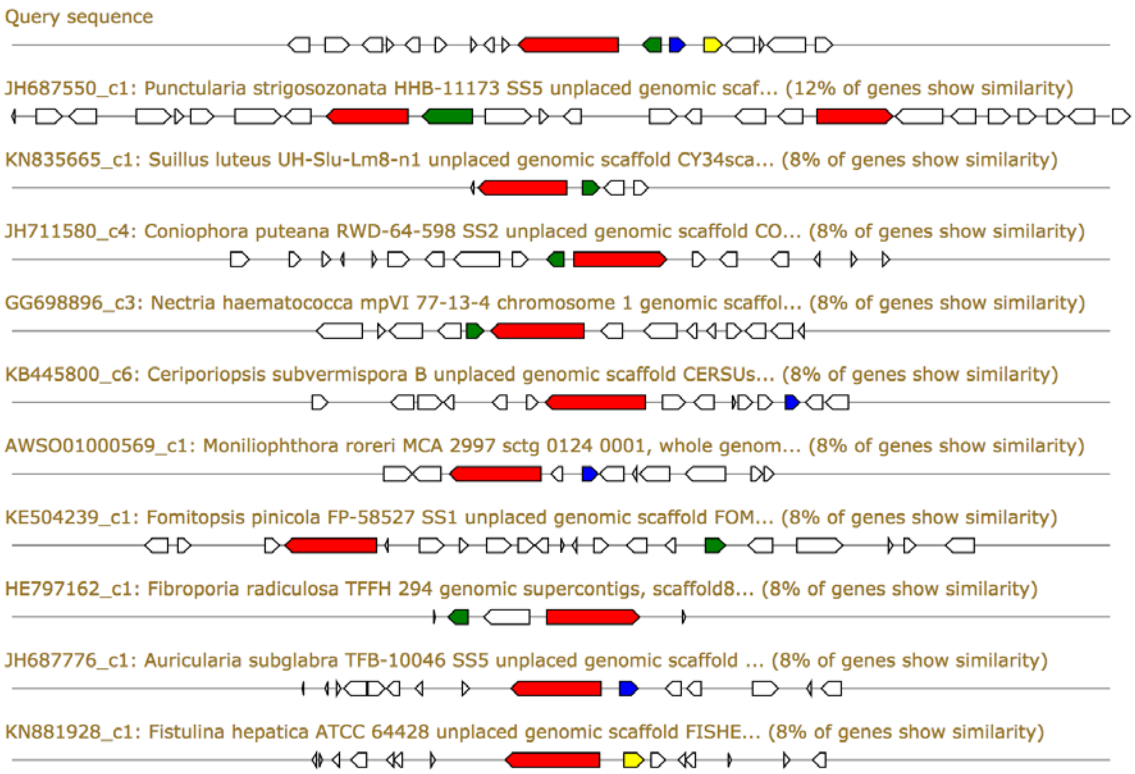
Query sequence
JH687550_c1: Punctularia strigosozonata HHB-11173 SS5 unplaced genomic scaf... (12% of genes show similarity)
KN835665_c1: Suillus luteus UH-Slu-Lm8-n1 unplaced genomic scaffold CY34sca... (8% of genes show similarity)
JH711580_c4: Coniophora puteana RWD-64-598 SS2 unplaced genomic scaffold CO... (8% of genes show similarity)
GG698896_c3: Nectria haematococca mpVI 77-13-4 chromosome 1 genomic scaffol... (8% of genes show similarity)
KB445800_c6: Ceriporiopsis subvermispora B unplaced genomic scaffold CERSUs... (8% of genes show similarity)
AWSO01000569_c1: Moniliophthora roreri MCA 2997 sctg 0124 0001, whole genom... (8% of genes show similarity)
KE504239_c1: Fomitopsis pinicola FP-58527 SS1 unplaced genomic scaffold FOM... (8% of genes show similarity)
HE797162_c1: Fibroporia radiculosa TFFH 294 genomic supercontigs, scaffold8... (8% of genes show similarity)
JH687776_c1: Auricularia subglabra TFB-10046 SS5 unplaced genomic scaffold ... (8% of genes show similarity)
KN881928_c1: Fistulina hepatica ATCC 64428 unplaced genomic scaffold FISHE... (8% of genes show similarity)

Supplementary Fig. S4

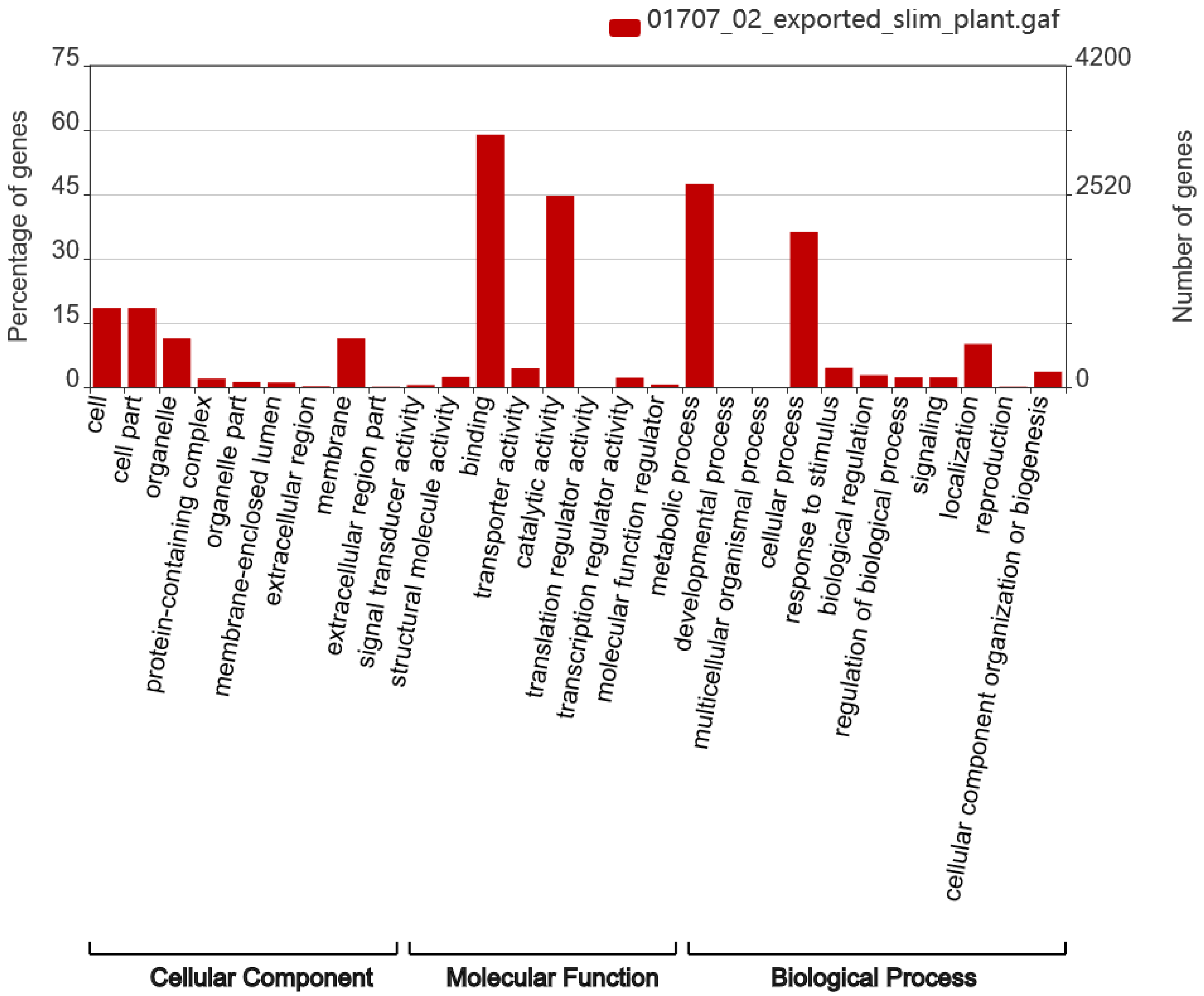

Supplementary Fig. S5

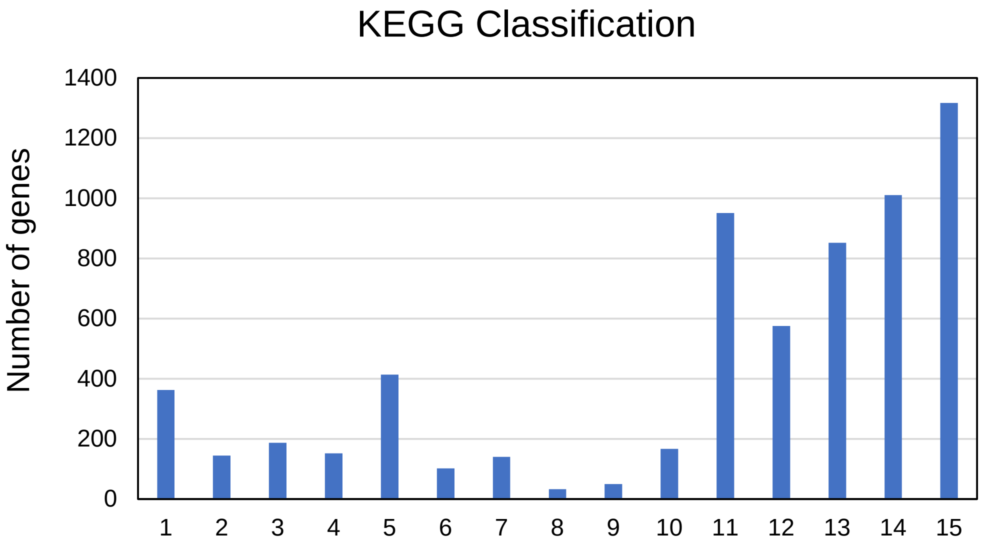

1. Carbohydrate metabolism (Metabolism)
2. Energy metabolism (Metabolism)
3. Lipid metabolism (Metabolism)
4. Nucleotide metabolism (Metabolism)
5. Amino acid metabolism (Metabolism)
6. Glycan biosynthesis/metabolism (Metabolism)
7. Metabolism of cofactors/vitamins (Metabolism)
8. Metabolism of terpenoids/polyketides (Metabolism)
9. Biosynthesis of other secondary metabolites (Metabolism)
10. Xenobiotics biodegradation/metabolism (Metabolism)
11. Genetic Information Processing
12. Environmental Information Processing
13. Cellular Processes
14. Organismal Systems
15. Diseases

Table S1. **Assembly statistics.**

Contig total number	32
Contig size (bp)	39,020,349
Contig mean length (bp)	1,219,386
Contig maximum length (bp)	4,666,114
Contig minimum length (bp)	23,725
Contig L50	5
Contig N50 (bp)	3,179,637
Contig L90	15
Contig N90 (bp)	1,017,009

Table S2. Gene model statistics.

Genes Total	13,157
Average Gene length (bp)	1,669.3
Gene Size (bp)	17,446,980
Transcripts Total	13,157
Average Transcripts length (bp)	1,326.1
Exons per genes	5.7
Protein Total	13,157
Average Protein length (bp)	441.0

Table S4. **Resources of the other 25 fungi for OrthoMCL analysis**

Abbreviation	Species	Downloading website
abis	*Agaricus bisporus*	http://genome.jgi.doe.gov/Agabi_varbisH97_2/Agabi_varbisH97_2.home.html
anig	*Aspergillus niger*	http://genome.jgi.doe.gov/Aspni_DSM_1/Aspni_DSM_1.home.html
ccin	*Coprinopsis cinerea*	http://www.broadinstitute.org/annotation/genome/coprinus_cinereus/MultiHome.html
cneg	*Cryptococcus neoformans*	http://www.broadinstitute.org/annotation/genome/cryptococcus_neoformans/MultiDownloads.html
cpar	*Cryphonectria parasitica*	http://genomeportal.jgi-psf.org/Crypa2/Crypa2.home.html
cput	*Coniophora puteana*	http://genome.jgi.doe.gov/Conpu1/Conpu1.home.html
dsqu	*Dichomitus squalens*	http://genome.jgi.doe.gov/Dicsq1/Dicsq1.home.html
glux	*Gymnopus luxurians*	http://genome.jgi.doe.gov/Gymlu1/Gymlu1.home.html
gtra	*Gloeophyllum trabeum*	http://genome.jgi.doe.gov/Glotr1_1/Glotr1_1.home.html
lbic	*Laccaria bicolor*	http://genome.jgi-psf.org/Lacbi2/Lacbi2.home.html
ledo	*Lentinula edodes*	http://legdb.chenlianfu.com/
mror	*Moniliophthora roreri*	http://www.ncbi.nlm.nih.gov/Traces/wgs/?val=AWSO01
ncra	*Neurospora crassa*	http://www.broadinstitute.org/annotation/genome/neurospora/MultiDownloads.html
pchr	*Phanerochaete chrysosporium*	http://genome.jgi.doe.gov/Phchr2/Phchr2.home.html
post	*Pleurotus ostreatus*	http://genome.jgi-psf.org/PleosPC15_2/PleosPC15_2.home.html
ppla	*Postia placenta*	https://genome.jgi.doe.gov/PosplRSB12_1/PosplRSB12_1.home.html
psti	*Pichia stipitis*	http://genome.jgi-psf.org/Picst3/Picst3.home.html
scer	*Saccharomyces cerevisiae*	http://downloads.yeastgenome.org/sequence/S288C_reference/orf_protein/
scom	*Schizophyllum commune*	http://genome.jgi-psf.org/Schco3/Schco3.home.html
slac	*Serpula lacrymans*	http://genome.jgi.doe.gov/SerlaS7_9_2/SerlaS7_9_2.home.html
snod	*Stagonospora nodorum*	http://genome.jgi.doe.gov/Stano2/Stano2.home.html
tree	*Trichoderma reesei*	http://genome.jgi-psf.org/Trire2/Trire2.home.html
tver	*Trametes versicolor*	http://genome.jgi.doe.gov/Trave1/Trave1.home.html

umay	*Ustilago maydis*	http://www.broadinstitute.org/annotation/genome/ustilago_maydis/Home.html
vvol	*Volvariella volvacea*	http://genome.jgi.doe.gov/Volvo1/Volvo1.home.html

Table S5. The loci of matA and matB genes.

	Gene	ID	Scaffold	Strand	Start	End	Protein length (aa)
matA-related genes							
	HD1	evm_000000F|arrow.597.2	000000F|arrow	-	1768661	1770872	665
	HD2	evm_000000F|arrow.598	000000F|arrow	+	1771134	1773073	595
	mip	evm_000000F|arrow.599	000000F|arrow	-	1773895	1776400	776
matB genes							
	sc_pr1	evm_000009F|arrow.285	000009F|arrow	+	807358	808999	456
	sc_pr2	evm_000009F|arrow.286	000009F|arrow	-	809633	811247	467
	sc_pp1	pp1	000009F|arrow	-	812739	812882	48
	sc_pr3	evm_000009F|arrow.287	000009F|arrow	-	813657	815134	405
	sc_pr4	evm_000009F|arrow.288	000009F|arrow	+	815483	816973	412
	sc_pr5	evm_000009F|arrow.289	000009F|arrow	-	817406	818732	334
	sc_pp2	pp2	000009F|arrow	+	819334	819462	43
	sc_pp3	evm_000009F|arrow.290	000009F|arrow	+	821494	821676	61
	sc_pr6	evm_000009F|arrow.293	000009F|arrow	+	828813	830599	557
	sc_pr7	evm_000009F|arrow.298	000009F|arrow	+	844144	845807	466

Table S7. **Identification of type I and type II β-glucan synthase genes in *S. crispa*.**

S. crispa gene	Gene ID	Identity (matched nucleotide/all)
ScrFKS1 (Type I)		
	EKM78523	79%(1415/1795)
	XP_001878782	82%(1470/1794)
	XP_001837755	79%(1417/1793)
	XP_003031402	84%(1357/1623)
	ADX07311	80%(1372/1709)
	EGO01123	82%(1466/1778)
	EIW79670	80%(1430/1783)
	EIM87404	81%(1444/1789)
	EIN14554	81%(1457/1788)
	EKM57278	87%(1553/1788)
	EMD36733	88%(1563/1785)
	CCM04495	90%(1598/1784)
	EIW53177	86%(1543/1789)
	EJF59836	87%(1552/1787)
	EJD01928	80%(1352/1700)
	EJU00969	77%(1387/1802)
ScrFKS2 (Type II)		
	EJD05928	80%(1368/1702)
	KJ187309	92%(1536/1666)
	EMD38542	90%(1486/1644)
	CCL99114	85%(1501/1759)
	EKM55441	84%(1483/1760)
	EIW60373	89%(1470/1643)
	EJF65840	88%(1450/1643)
	EIN07381	83%(1370/1646)
	EIW85198	81%(1430/1769)
	EIM90013	82%(1437/1763)
	XP_003035758	79%(1402/1767)
	XP_001833273	79%(1403/1766)
	XP_001875386	77%(1271/1648)
	EKM82691	85%(1394/1644)

Table S8. **List of biosynthetic gene clusters in the S. crispa genome.**

	Gene ID	Gene start	Gene end	Strand	Gene description	EC number
contig_000000F - Gene Cluster 1. Type = other. Location: 298010 - 342192 nt.	SCP_0101000	318009	322192	-	SMCOG1002:AMP-dependent_synthetase_and_ligase	
contig_000000F - Gene Cluster 2. Type = other. Location: 2151671 - 2195752 nt.	SCP_0107660	2171670	2175752	+	SMCOG1002:AMP-dependent_synthetase_and_ligase	
contig_000001F - Gene Cluster 3. Type = terpene. Location: 577100 - 598315 nt.	SCP_0201800	587099	588315	+	SMCOG1052:Terpene_synthase/cyclase_metal-binding_domain_protein	
contig_000001F - Gene Cluster 4. Type = other. Location: 1226000 - 1279843 nt.	SCP_0203810	1245999	1259843	-	SMCOG1002:AMP-dependent_synthetase_and_ligase	
contig_000002F - Gene Cluster 5. Type = terpene. Location: 1420916 - 1442540 nt.	SCP_0304660	1430915	1432540	+	SMCOG1052:Terpene_synthase/cyclase_metal-binding_domain_protein	
contig_000002F - Gene Cluster 6. Type = t1pks. Location: 1903728 - 1956364 nt.	SCP_0306490	1932910	1936364	+	SMCOG1002:AMP-dependent_synthetase_and_ligase	
	SCP_0306480	1923727	1930460	-	SMCOG1022:Beta-ketoacyl_synthase	EC:3.1
contig_000002F - Gene Cluster 7. Type = terpene. Location: 2394718 - 2415961 nt.	SCP_0308030	2404717	2405961	-	SMCOG1052:Terpene_synthase/cyclase_metal-binding_domain_protein	
contig_000003F - Gene Cluster 8. Type = terpene. Location: 780278 - 801362 nt.	SCP_0403040	790277	791362	-	SMCOG1052:Terpene_synthase/cyclase_metal-binding_domain_protein	
contig_000003F - Gene Cluster 9. Type = terpene. Location: 1471027 - 1501332 nt.	SCP_0405790	1490111	1491332	+	TRI5	EC:4.2.3.6
	SCP_0405760	1481026	1482251	+	TRI5	EC:4.2.3.6
contig_000003F - Gene Cluster 10. Type = terpene. Location: 1523295 - 1544372 nt.	SCP_0405960	1533294	1534372	-	TRI5	EC:4.2.3.6
contig_000004F - Gene Cluster 11. Type = other. Location: 2837290 - 2881205 nt.	SCP_0509750	2857289	2861205	-	SMCOG1002:AMP-dependent_synthetase_and_ligase	
contig_000005F - Gene Cluster 12. Type = indole. Location: 730480 - 752441 nt.	SCP_0602770	740479	742441	-	indole_PTase	EC:2.5.1
contig_000005F - Gene Cluster 13. Type = terpene. Location: 1217614 - 1239213 nt.	SCP_0604400	1227613	1229213	+	SMCOG1052:Terpene_synthase/cyclase_metal-binding_domain_protein	
contig_000005F - Gene Cluster 14. Type = other. Location: 1681418 - 1725375 nt.	SCP_0605700	1701417	1705375	+	SMCOG1002:AMP-dependent_synthetase_and_ligase	
contig_000006F - Gene Cluster 15. Type = terpene. Location: 344484 - 365726 nt.	SCP_0701300	354483	355726	-	phytoene_synt	EC:2
contig_000007F - Gene Cluster 16. Type = terpene. Location: 1818743 - 1839963 nt.	SCP_0806170	1828742	1829963	+	SMCOG1052:Terpene_synthase/cyclase_metal-binding_domain_protein	
contig_000008F - Gene Cluster 17. Type = terpene. Location: 106419 - 130408 nt.	SCP_0900460	119213	120408	+	TRI5	EC:4.2.3.6
	SCP_0900450	116418	117708	+	TRI5	EC:4.2.3.6
contig_000008F - Gene Cluster 18. Type = other. Location: 882452 - 926420 nt.	SCP_0903030	902451	906420	-	SMCOG1002:AMP-dependent_synthetase_and_ligase	
contig_000008F - Gene Cluster 19. Type = terpene. Location: 1136595 - 1159210 nt.	SCP_0903760	1146594	1149210	+	terpene_cyclase	EC:5.4
contig_000008F - Gene Cluster 20. Type = terpene. Location: 1472478 - 1494300 nt.	SCP_0905140	1482477	1484300	-	phytoene_synt	EC:2.5.1.21

contig_000011F - Gene Cluster 21. Type = other. Location: 351494 - 398797 nt.					
SCP_1101170	365576	368220	-	SMCOG1002:AMP-dependent_synthetase_and_ligase	
SCP_1101190	371493	378797	-	SMCOG1127:condensation_domain-containing_protein	EC:1.2.1.31
SCP_1101230	385659	387304	+	SMCOG1010:NAD-dependent_epimerase/dehydratase	EC:1.1.1.145
SCP_1101200	379431	380563	-	SMCOG1030:serine/threonine_protein_kinase	
SCP_1101160	363311	364787	+	SMCOG1030:serine/threonine_protein_kinase	
contig_000010F - Gene Cluster 22. Type = t1pks. Location: 885689 - 934146 nt.					
SCP_1203090	905688	914146	+	SMCOG1022:Beta-ketoacyl_synthase	
contig_000010F - Gene Cluster 23. Type = indole-nrps. Location: 1041798 - 1097259 nt.					
SCP_1203670	1081255	1083237	-	SMCOG1034:cytochrome_P450	EC:1.14
SCP_1203620	1061797	1063417	-	dmat	EC:2.5.1
SCP_1203640	1067899	1077259	-	SMCOG1002:AMP-dependent_synthetase_and_ligase	
SCP_1203600	1055515	1057744	-	SMCOG1034:cytochrome_P450	EC:1.14
contig_000012F - Gene Cluster 24. Type = terpene. Location: 174101 - 201152 nt.					
SCP_1300670	190035	191152	+	SMCOG1052:Terpene_synthase/cyclase_metal-binding_domain_protein	
SCP_1300680	191672	193134	-	SMCOG1036:alpha/beta_hydrolase_fold_protein	EC:2.3.1
SCP_1300650	184100	185284	+	SMCOG1052:Terpene_synthase/cyclase_metal-binding_domain_protein	
contig_000013F - Gene Cluster 25. Type = terpene. Location: 466945 - 488536 nt.					
SCP_1401670	476944	478536	-	SMCOG1052:Terpene_synthase/cyclase_metal-binding_domain_protein	
contig_000014F - Gene Cluster 26. Type = terpene. Location: 887958 - 909591 nt.					
SCP_1503070	897957	899591	-	SMCOG1052:Terpene_synthase/cyclase_metal-binding_domain_protein	
contig_000015F - Gene Cluster 27. Type = t1pks. Location: 462780 - 507320 nt.					
SCP_1601610	464481	466711	+	SMCOG1034:cytochrome_P450	EC:1.14
SCP_1601690	482779	487320	+	SMCOG1022:Beta-ketoacyl_synthase	EC:3.1
contig_000016F - Gene Cluster 28. Type = other. Location: 119822 - 164105 nt.					
SCP_1700600	139821	144105	+	SMCOG1002:AMP-dependent_synthetase_and_ligase	
contig_000016F - Gene Cluster 29. Type = t1pks. Location: 567660 - 616617 nt.					
SCP_1702150	598219	599487	+	SMCOG1036:alpha/beta_hydrolase_fold_protein	
SCP_1702140	587659	596617	+	SMCOG1022:Beta-ketoacyl_synthase	
contig_000016F - Gene Cluster 30. Type = t1pks. Location: 678964 - 727930 nt.					
SCP_1702470	678174	680138	-	SMCOG: SMCOG1030 (serine/threonine protein kinase)	
SCP_1702560	698963	707930	-	SMCOG1022:Beta-ketoacyl_synthase	
